# Building living systematic reviews and reporting standards for comparative microscopic analysis of white diseases in hard corals

**DOI:** 10.1002/ece3.11616

**Published:** 2024-07-04

**Authors:** C. E. Page, E. Anderson, T. D. Ainsworth

**Affiliations:** ^1^ School of Biological, Earth and Environmental Sciences (BEES) University of New South Wales (UNSW) Kensington New South Wales Australia; ^2^ College of Science and Engineering Flinders University Bedford Park South Australia Australia

**Keywords:** coral disease, disease description, histopathology, living review, systematic review

## Abstract

Over the last 4 decades, coral disease research has continued to provide reports of diseases, the occurrence and severity of disease outbreaks and associated disease signs. Histology using systematic protocols is a gold standard for the microscopic assessment of diseases in veterinary and medical research, while also providing valuable information on host condition. However, uptake of histological analysis for coral disease remains limited. Increasing disease outbreaks on coral reefs as human impacts intensify highlights a need to understand the use of histology to date in coral disease research. Here, we apply a systematic approach to collating, mapping and reviewing histological methods used to study coral diseases with ‘white’ signs (i.e., white diseases) in hard coral taxa and map research effort in this field spanning study design, sample processing and analysis in the 33 publications identified between 1984 and 2022. We find that studies to date have not uniformly detailed methodologies, and terminology associated with reporting and disease description is inconsistent between studies. Combined these limitations reduce study repeatability, limiting the capacity for researchers to compare disease reports. A primary outcome of this study is the provision of transparent and repeatable protocols for systematically reviewing literature associated with white diseases of hard coral taxa, and development of recommendations for standardised reporting procedures with the aim of increasing uptake of histology in addition to allowing for ongoing comparative analysis through living systematic reviews for the coral disease field.

## Graphical abstract

Diseases and disease outbreaks are increasing on coral reefs. This study systematically reviews histological methods used to study white diseases in hard coral species from 1984 to 2022 and finds inconsistencies in reporting methodologies and terminology among studies. As a primary outcome, this study proposes standardised reporting procedures to standardise ongoing comparative analysis in the field.

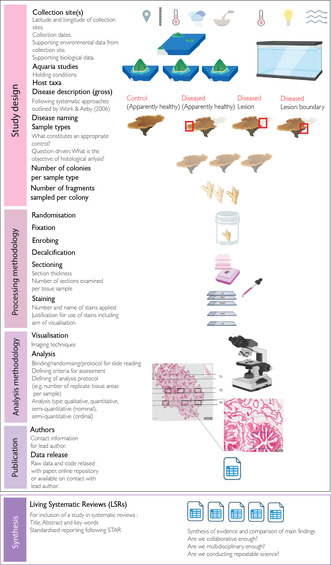

## 1 INTRODUCTION

Coral diseases pose an immediate threat to coral reef ecosystems because they can contribute to the reduction in abundance and cover of calcifying habitat‐forming corals (Estrada‐Saldívar et al., 2021; Heres et al., 2021; Miller et al., 2009; Precht et al., 2016), and ecosystem phase shifts (Harvell et al., 2007; Norström et al., 2009). Given the ongoing impact of climate change and anthropogenic stressors on coral reefs (MacNeil et al., 2019; Maynard et al., 2015; Sokolow, 2009) it is critical to develop and apply standardised tools to understand coral health and to allow for future comparative analysis. Histology is the study of cells and tissues using light or electron microscopy; histopathology is a subfield in the field of pathology that uses multiple methods to prepare tissue sections to be examined using light or electron microscopy for the diagnosis of diseases from cells and tissue lesions. Coral disease research has predominantly focused on field surveys (disease occurrence and prevalence within a reef ecosystem), gross pathological assessment (disease description without microscopic assessment) and bacteriology (Work & Meteyer, 2014). Assessment of disease at epidemiological scales is a rapid way to gain information on disease dynamics and impacts within a population, but provide no information on host condition, possible causes of lesions (disease aetiology) and other non‐visible impacts of stress (Galloway et al., 2007; Work & Meteyer, 2014).

**FIGURE 1:**
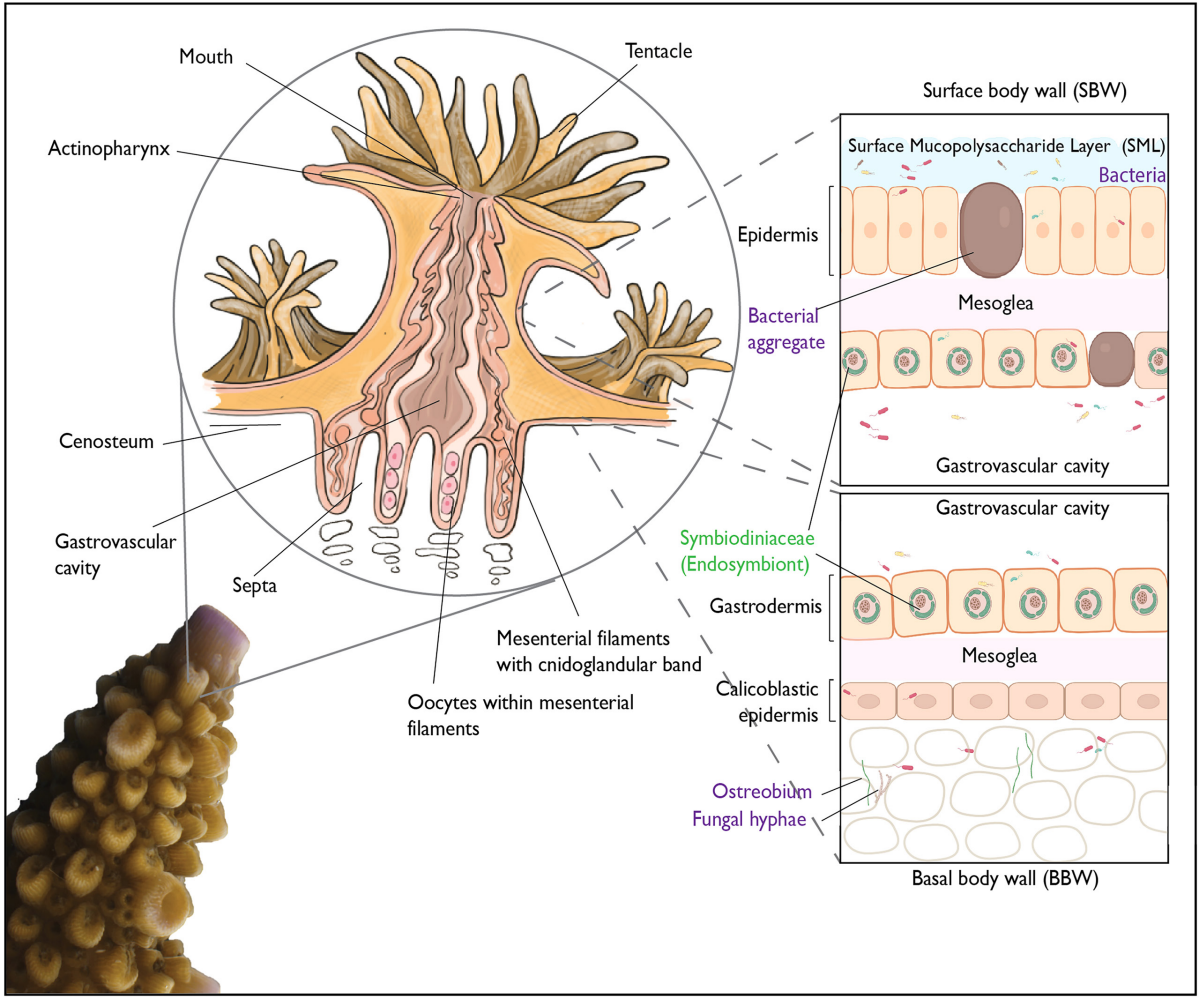
Diagram depicting the anatomy of a coral with tissue layers as viewed by histology.

Scleractinian corals (hard corals) are simple, multicellular organisms where interconnected polyps form the colonial organism. Individual polyps are formed from a surface body wall which provide the outer most barrier between the organism and its surroundings (Figure 1) and a basal body wall that anchors coral tissue to its skeleton (Figure 1) (Hawthorn et al., 2023; LaDouceur, 2021; Woodley et al., 2016).These tissue layers are made up of an epidermis, connective tissue termed the mesoglea, and gastrodermis which hosts the photoendosymbiotic dinoflagellates (Figure 1) (Hawthorn et al., 2023; LaDouceur, 2021; Woodley et al., 2016). The epidermis comprises of the epithelium with columnar cells, mucocytes and cnidocytes (specialised stinging cells) (Figure 1) (Hawthorn et al., 2023; LaDouceur, 2021; Woodley et al., 2016). The basal body wall is comprised of the epithelial calicodermis, desmocytes and mucocytes and is the layer responsible for secreting a soluble organic matrix facilitating calcium carbonate secretion (Figure 1) (Hawthorn et al., 2023; LaDouceur, 2021; Woodley et al., 2016).

Histological analysis of coral tissues involves fixation of coral colony fragments and decalcification to remove the network of calcium carbonate skeleton, tissues can then be processed with standard techniques to allow microscopic assessment of coral tissues and cell structure (Greene et al., 2020; Price & Peters, 2018). Histology has also been used to identify microbes, parasites (Aeby, Shore, et al., 2021; Work et al., 2021), assess reproductive state (Ward, 1995) and cellular assessment of endosymbiosis breakdown (bleaching) (Brown, 1997; Brown et al., 1995; Gates et al., 1992; Weis, 2008). Studies have also applied a variety of stains, imaging and analysis techniques to assess coral tissue integrity, thickness, cell death, necrosis and algal symbiont characteristics (Ainsworth et al., 2008; Ainsworth, Kramasky‐Winter, et al., 2007; Gierz et al., 2020; Meiling et al., 2021; Work & Rameyer, 2005). Histology is also key to marrying colony‐scale gross observations of disease lesions to coral condition (Ainsworth et al., 2008; Ainsworth, Kramasky‐Winter, et al., 2007; Ainsworth, Kvennefors, et al., 2007; Eaton et al., 2021). Often, differences at the cellular level are apparent before visible signs of stress manifest at the whole colony level (Ainsworth et al., 2008) and similar visual signs of diseases in corals can have very different pathologies at a tissue level (Ainsworth, Kramasky‐Winter, et al., 2007; Landsberg et al., 2020).

Despite increased risk of disease emergence on coral reefs and continued calls for establishment of standardised terminology and pathological tools (Galloway et al., 2007; Pollock et al., 2011; Weil et al., 2006; Work & Meteyer, 2014) uptake has remained slow (Work & Meteyer, 2014). Work and Meteyer (2014) called for increased multi‐disciplinarity to address a disconnect between those who study and elucidate the causes of animal disease (i.e., veterinary scientists and pathologists), and those who study coral biology and ecology. Doing so will address ambiguous communication of findings and a deficiency in consistent and comprehensive protocols within the literature.

Increasingly, there is a growing need for reliable synthesis of research to address challenges facing the environment. Now a gold standard in many fields, systematic reviews use transparent methodology to overcome problems of bias commonly associated with narrative reviews for summarising literature (Pussegoda et al., 2017). Systematic reviews are commonly used to inform practice, policy and management because they are assumed to be comprehensive and reproducible syntheses (Bilotta et al., 2014; Pullin & Stewart, 2006). Review methodology can be tailored for the research questions at hand and can vary in the depth of synthesis from mapping research areas, to rapid reviews or more comprehensive qualitative and quantitative protocols including meta‐analysis (Lagisz et al., 2022). Mapping research effort can help identify patterns and knowledge gaps in the current body of literature (Miake‐Lye et al., 2016). For example, a recent systematic study of coral reef literature concluded that there are prevalent biases present in representation of genders and non‐OECD (Organisation for Economic Co‐operation and Development) nations of authors (Ahmadia et al., 2021); and a systematic review of coral health literature found that countries in the Global South with large coral reef areas were underrepresented (Burke et al., 2023). Identifying these evidence ‘gaps’ can be indicative of barriers in research fields. Living systematic reviews are an emerging approach that involves continual updating of systematic reviews to include the most recent research with the aim of making relevant evidence available to users, including scientists and decisions makers (Khamis et al., 2019).

Here, we provide a systematic protocol for collating and reviewing studies that have used histology to microscopically investigate coral diseases with white disease signs (i.e., white diseases) in hard coral taxa over the past 4 decades. In this study, we use the term ‘white disease’ in reference to coral diseases characterised by white lesions of paling tissue or partial mortality leading to exposed white skeleton (tissue loss). Paling of coral tissue has been reported preceding tissue loss for multiple coral diseases, including stony coral tissue loss disease in the Caribbean (Aeby, Ushijima, et al., 2021; Meiling et al., 2021) and *Montipora* white syndrome in the Pacific (Page et al., 2023). Paling in association with disease can be differentiated from the whole coral colony response of coral paling and bleaching (Brown, 1997) based on the patchy, focal or multi‐focal extent of lesions (Work & Aeby, 2006). Coral diseases involving white disease signs have been reported as some of the most damaging and widespread diseases on coral reefs and include white plague, white‐band disease and white syndromes (Bourne et al., 2015; Bythell et al., 2004; Willis et al., 2004) that all can cause significant coral mortality (Aronson & Precht, 2001; Estrada‐Saldívar et al., 2021; Heres et al., 2021; Willis et al., 2004). While there is a growing knowledge base of the biology and ecology of several other tissue loss diseases like black band (Sato et al., 2016) and brown band (Bourne et al., 2008; Lobban et al., 2011), there is still much to be learned about white diseases in hard coral taxa.

The primary research question addressed in this study is:

What histological methods have studies used to microscopically assess white diseases in hard coral taxa?

Secondary questions we also address in this review are:

- What is the research effort in this field?
- What is the study design of publications that have used histology to investigate disease?
- What methods have publications used in preparation, processing and assessing histology?
- What is the standard of reporting for methodology of publications?

In doing this, we hope to provide (1) transparent and repeatable protocols for systematically reviewing literature associated with white diseases of hard coral taxa, and (2) standard reporting recommendations for future coral disease studies to aid uptake and continued synthesis and appraisal of research through establishing living review procedures.

## 2 METHODS

### 2.1 Systematic review

We conducted a systematic identification of literature using histological methods to assess white diseases in hard coral taxa. This review protocol follows the reporting guidelines set out by the PRISMA‐P (Preferred Reporting Items for Systematic Reviews and Meta‐Analyses—Protocols) (Shamseer et al., 2015) and is amended with the reporting standards of ROSES (Reporting standards for Systematic Evidence Syntheses) (Haddaway et al., 2018). A conceptual diagram showing systematic protocol development is detailed in Figure S1.

Specifically, we used an initial pilot phase to identify relevant key words, exclusion and inclusion criteria, in addition to defining a detailed search and data collection plan (Appendix S1, Figure S1). Scopus was selected for this study as it offers high coverage of subjects relevant to life sciences (Mongeon & Paul‐Hus, 2016). Search terms included those referring to organism (hard corals), disease (white diseases and tissue loss) and method (histology) (Table 1). The final list of search terms for application in academic data bases Scopus and Web of Science were applied on the 24th February 2023 (Table 1). The returned lists of references in each database were exported into reference management software Zotero (Vanhecke, 2008), and reference lists were merged and duplicates removed.

**TABLE 1:** Final search terms used for systematic review of the literature.

Category	Key word
Organism	“coral*” OR “scleractini*” OR “cnidaria*” “Hard coral”
Disease reference	“white‐band” OR “white band” OR “tissue loss” OR “white syndrome” OR “white disease” OR “SCTL*” OR “stony coral tissue loss” OR “plague”
Method	“histo*”

The pilot screening phase developed exclusion and inclusion criteria to identify literature for full‐text review (see Figure S1, Appendix S1). The final criteria (Table 2) formed a decision tree, which was then applied to all studies returned by the searches and were carried out by a single reviewer (CP) in Rayyan (www.rayyan.ai). A final search was completed in Google Scholar to screen for any relevant publications not returned through Scopus or Web of Science. To do this, inclusion and exclusion criteria were applied to the first 10 pages returned on seven search strings by a single reviewer (CP): “coral histo* stony coral tissue loss*”, “coral histo* white syndrome”, “coral histo* plague”, “coral histo* white band” and “coral histo* lesion”.

**TABLE 2:** Final inclusion and exclusion criteria for screening of the literature.

Inclusion criteria	The study likely considers hard coral taxa
	The study likely considers a coral disease with white signs
	The study likely conducts histological analysis
	The study is in English
	The study is peer‐reviewed
Exclusion criteria	The study likely refers to some other disease type that does not involve tissue loss (e.g., growth anomaly)
	The study likely refers to some other disease type that involves other disease signs that are not white diseases (black band, brown band, pink spots etc.)
	The study is secondary (i.e., a review of the literature)

For each of the publications included in this study, we systematically recorded information (i.e., data extraction) through filling in a pre‐coded data sheet in GoogleForms for general data extraction (see https://forms.gle/4enApHx2qtJMeQX5A, Appendix S1) and a quality of methods reporting appraisal (see https://forms.gle/t3s4L35LUkXc93um7, Appendix S1). Data categories followed the research questions presented in the introduction. Our approach followed PICO (Population or Problem, Intervention or Exposure, Comparison and Outcome) elements commonly used in evidence synthesis (James et al., 2016). The functioning of the datasheet was piloted on 10 randomly selected articles. Data extraction allowed mapping of research effort through collected information on first, second and last authors of publications including country of institution (further classified as low‐ and middle‐income countries) (OECD, 2020) and when stated online (through self‐report) pronouns used following guidelines for gender outlined by SAGER (i.e., he/she/them; Heidari et al., 2016) and year of publication.

Information was collected on study scope, including location of the study, study type (field or experimental), diseases studied, taxa studied and other methods utilised unrelated to histopathology. Location of the study was classified as the primary region of coral sample collection and grouped into major biogeographical regions (as per Crisp et al., 2022). We extracted data for fields relevant to histological methods and sample preparation including information on sample types, decalcification, embedding, staining and imaging. We extracted data on methods for histological analysis including the type of data collected (i.e., qualitative, quantitative or semi‐quantitative), and specific tissue structures/conditions/agents that were examined. Additional comment fields were used to capture relevant information including the main findings of each study.

We used the STAR (Structure, Transparent, Accessible Reporting) methods protocol outlined by Cell Journal (Marcus, 2016) in addition to work by Gibson‐Corley et al. (2013) presenting best practice for scoring histopathological tissues to develop criteria in which to appraise the quality of methods reporting in each of the studies. General instructions for STAR methods include reporting methods in sufficient detail, so readers do not need to refer to other papers to understand how procedures were performed. The developed criteria consisted of marking studies based on categories: resource availability, method details and quantification and statistical analysis. Here we also collect information on reproducibility of slide assessment (following definition as per Gundersen, 2021) in addition to reporting of sample sizes. Table S1 presents criteria for data extraction for methods reporting appraisal.

### 2.2 Data visualisation and statistical analyses

We used R version 4.1.1 (2021‐08‐10) (R Development Core Team, 2010) for analyses and data visualisation. All code and raw data are available at https://github.com/CharlotteEPage/Histology_methods_systematic_review.git. When multiple methods and/or data fields were used in a study, each category was counted independently for summary statistics and visualisation. Diseases studied were grouped hierarchically by the affected taxa and the disease name (e.g., *Montipora* white syndrome). If a study included investigation of multiple species and diseases each combination was separately accounted for. Spatial illustrations were made using Google My Maps (Google, 2023) to record latitude and longitude of sampling regions and QGIS version 3.26 (QGIS Development Team, 2022). World map was downloaded from Natural Earth (https://www.naturalearthdata.com/) and coral reef locations from UNEP‐WCMC (https://data.unep‐wcmc.org/datasets/1) (UNEP‐WCMC et al., 2021).

## 3 RESULTS

### 3.1 Systematic identification of literature

Searches of Scopus and Web of Science using the final search terms yielded 71 and 162 articles respectively. After removal of 59 duplicates, 174 articles were screened on Rayyan. Thirty articles were deemed relevant to this study and included for full‐text screening. One hundred forty‐four articles were excluded from the study because they were not in English, did not use histology, did not consider hard coral or considered other coral diseases. Five articles were further deemed relevant for this study through Google Scholar searches, leading to a total of 35 papers reviewed at full‐text for data extraction. At full‐text review, a further two publications were removed from the review because they used only transmission electron microscopy methods, which is out of scope for this review. This left a final list of 33 publications for data extraction. A ROSES flow chart detailing results of our systematic protocol can be found in Figure S2.

### 3.2 Overview of systematic review data

**FIGURE 2:**
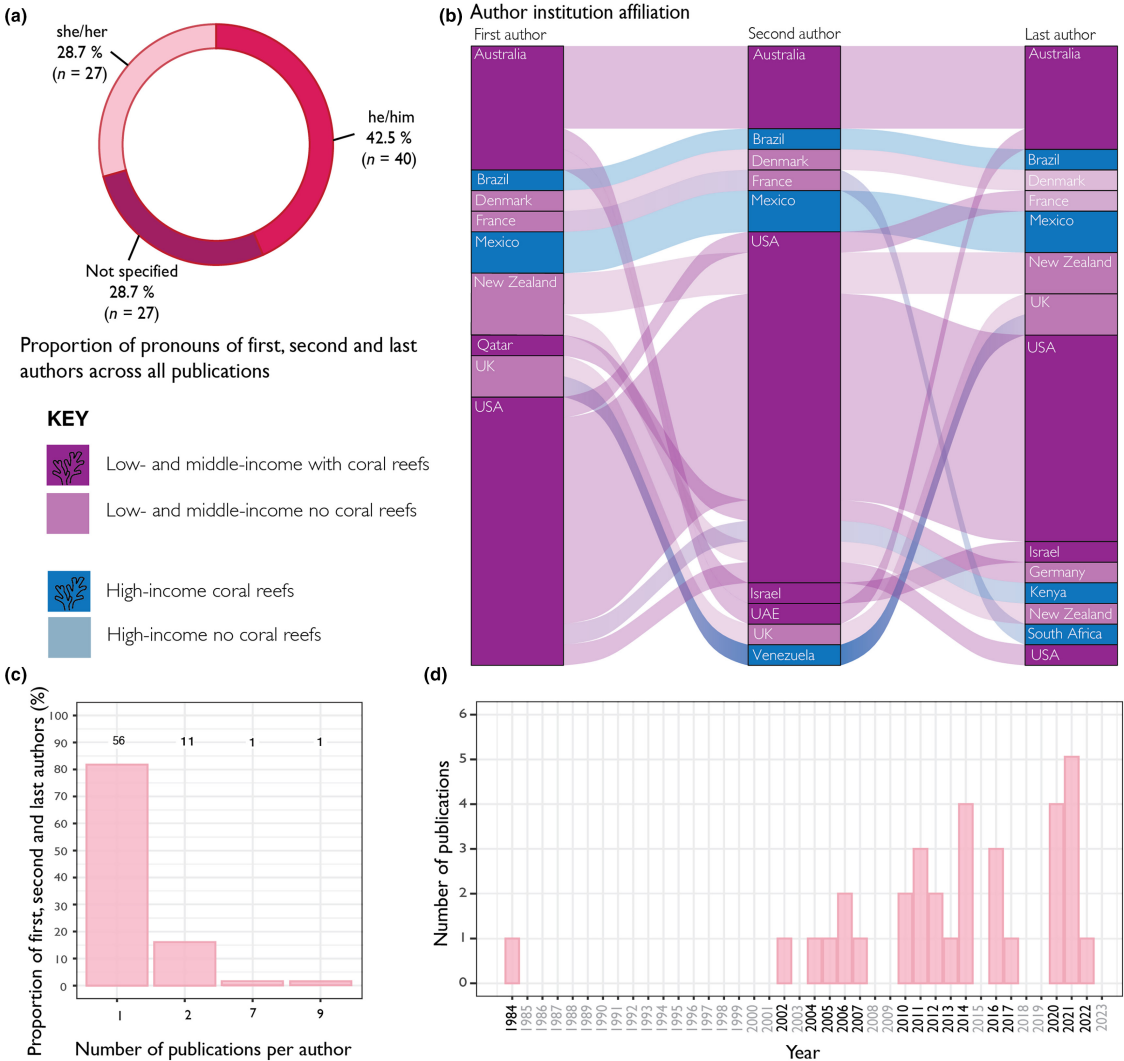
(a) Gender of first, second and last authors. (b) Sankey diagram of studies with more than two authors depicting collaborations between countries of author affiliation institution. Countries with coral reefs are shaded darker than those without coral reefs. (c) The total number of publications co‐authored by first, second and last authors. Numbers above bars represent counts. (d) The total number of publications included within this review in each year. Years in grey had no publications relevant to this review.

Of the 33 studies included in this review, 28.7% (*n* = 27) of first, second and last authors were identified as female and 42.5% (*n* = 40) were identified as male (Figure 2a). The remaining authors (28.7%, *n* = 27) did not clearly have gender specified online (Figure 2a). Of the 27 first, second and last authors with no gender identified, 10 authors were from countries classified as low‐ and middle‐income countries. Of the last authors only, 57.6% (*n* = 19) were identified as male, 18.2% (*n* = 6) identified as female and 24.2% (*n* = 8) did not clearly have gender specified online. First, second and last authors were affiliated with 16 countries. In total, the highest proportion of authors were affiliated with institutions in the United States (46.8%, *n* = 44), followed by Australia (16%, *n* = 15) (Figure 2b). Mexico and New Zealand had the same representation of authors (6.4%, *n* = 6) (Figure 2b). The remaining countries were affiliated with five or less authors. In total, 67% (*n* = 63) of authors were affiliated with institutions in countries classified as high‐income countries with coral reefs, 19% (*n* = 18) from high‐income countries with no coral reefs, and 13.8% (*n* = 13) from low‐ and middle‐income countries with coral reefs (Figure 2b). We recorded no instances of authors with affiliations from low‐ and middle‐income countries with no coral reefs.

Of those studies that had more than two authors (*n* = 29), authors with affiliation at institutions in Australia, United States and Denmark commonly formed collaborations between first, second and last authors (Figure 2b). Authors with affiliation at institutions in developing countries with coral reefs (i.e., Mexico and Brazil) formed within country collaborations, whereas authors in institutions in Venezuela, South Africa and Kenya formed collaborations with authors in the United States, United Kingdom and France (Figure 2b). 82% (*n* = 56) of first, second and last authors co‐authored a single paper (Figure 2c). A single author each co‐authored seven and nine publications respectively (Figure 2c). The 33 studies included in the review ranged in publication years from 1984 to 2022 (Figure 2d). There is a steady increase in the number of studies published over time following a gap in relevant publications from 1985 to 2002 (Figure 2d). There is a spike in publications using histological methods in 2021, where a maximum of five relevant publications were returned (Figure 2d).

### 3.3 Scope of studies

**FIGURE 3:**
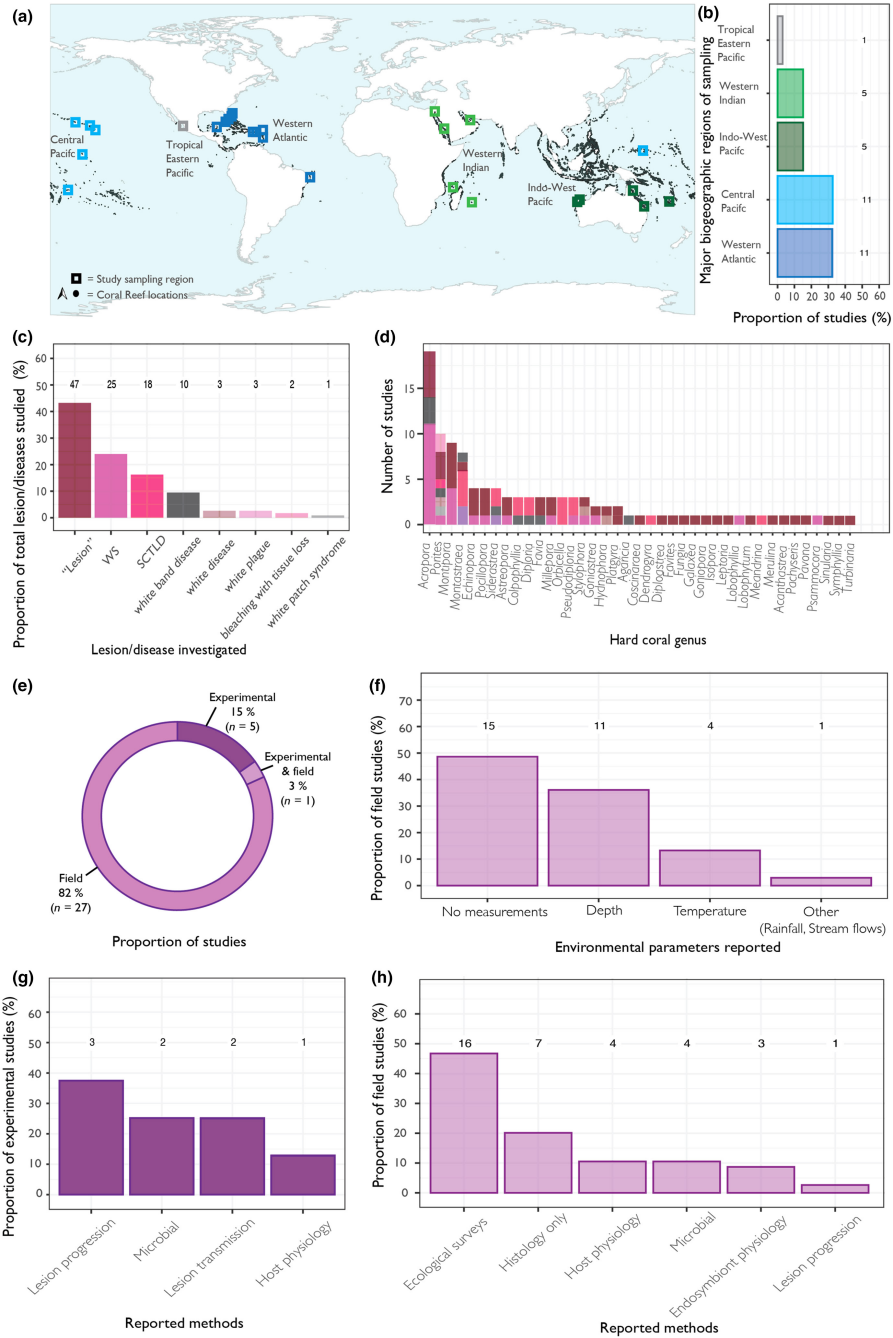
(a) Map showing major biogeographic regions of sampling of coral colonies for histology by studies. Squares represent general study sampling regions. Dark points represent coral reef locations downloaded from UNEP‐WCMC (https://data.unep‐wcmc.org/datasets/1). (b) Proportion of studies that sampled colonies for histology in each major biogeographic region. Numbers above bars indicate publication counts. (c) Diseases studied plotted as a proportion of the total diseases (genera, disease combination) recorded in this review. Numbers above bars represent counts. (d) The number of studies that focused on genera. Bars are coloured by disease name, with colours matching those associated with disease names in plot (c). (e) Proportion of studies that have used histology to investigate disease in samples taken from the field (in situ at a reef site) or experimentally (samples were taken from in situ and placed ex situ where corals were kept in aquaria before samples were taken). (f) Proportion of studies conducted in the field that reported other environmental parameters at reef sites. Numbers above bars indicate publication counts. (g) Proportion of experimental studies that reported additional methods to study disease. Numbers above bars indicate publication counts. (h) Proportion of field studies that reported additional methods to study disease. Numbers above bars indicate publication counts.

In total, 32 of the 33 included studies specified the region of sampling of coral colonies for histology analysis (Figure 3a,b). The single study that did not specify a sampling location was experimental. Studies represent sampling locations across all major biogeographic regions with coral reefs (Figure 3a,b). A single study included sampling locations of corals from two distinct bioregions (Indo‐West Pacific and Central Pacific). Eleven studies examined corals sampled from Western Atlantic (Figure 3a,b), five of these studies were based in a single state of the United States (Florida), and the remaining studies were located on Caribbean reefs including US Virgin Islands, Virgin Islands, Dominica and a single study was located on a Brazilian reef (Ponta do Seixas) (Figure 3a). Eleven studies studied corals sampled from Central Pacific (Figure 3a,b), including three studies located in a single offshore state of the United States, Hawaii and other sampling locations were American Samoa, Micronesia, New Caledonia and Palmyra Atoll National Wildlife Refuge (Figure 3a,b). A further five studies (15%) were in the Indo‐West Pacific and the Western Indian Ocean, respectively. Studies in the Indo‐West Pacific included Australian coral reefs, Heron Island and surrounding reefs and Lizard Island on the Great Barrier Reef, in addition to Montebello and Barrow Island in Western Australia (Figure 3b). Two of studies located in the Western Indian Ocean sampled corals in the Red Sea and one in the Persian Gulf (Figure 3a). Two studies sampled corals from coral reefs on islands surrounding Madagascar (Mayotte, Reunion) (Figure 3a). A single study was recorded as sampling corals from the Tropical Eastern Pacific and Mexican Pacific (Figure 3a,b).

Studies included seven separately named diseases or disease states: bleaching with tissue loss, stony coral tissue loss disease (SCTLD), white band, white disease, white patch, white plague and white syndrome (WS), in addition to reference to study of a lesions only with no specific disease specified (Figure 3c). In total, studies assessed 109 diseases, taxa combinations. The most highly cited change in corals investigated was ‘Lesion’ (43%, *n* = 47), followed by WS (23%, *n* = 25) and SCTLD (16.5%, *n* = 18) (Figure 3a). In total, 39 hard coral genera were studied (Figure 3d). The most studied genera were *Acropora*, *Porites* and *Montipora* (Figure 3d). Twenty one genera were studied in the context of a single disease name (Figure 3d). Studies ranged in the number of species included from 1 to 52. Sixteen studies focused on a single species.

In total, 82% (*n* = 27) of publications investigated coral diseases in the field (i.e., samples were taken in situ at a reef site) and 15% (*n* = 5) investigated disease in an experimental context (i.e., samples were taken from in situ and placed ex situ where corals were kept in aquaria before samples were taken) (Figure 3e). A single publication investigated coral disease in the field, and in an experimental context (Figure 3e). Of the 27 studies that investigated coral disease in the field, 15 did not report on any environmental variables taken at the site of collection, 11 studies reported depth and four studies reported temperature (Figure 3f). A single study reported other environmental information of rainfall and stream flows. Experimental studies included use of other methods to investigate disease including lesion progression, lesion transmission (i.e., exposure of an apparently healthy coral to a coral sample showing one or more lesions), analysis of microbial communities and host physiology (Figure 3g). Studies that investigated coral disease in the field also used other methods including ecological surveys, measures of host physiology, measures of endosymbiont physiology, microbial community analysis, lesion progression and testing of disease treatments (Figure 3h). 20% (*n* = 7) of field studies used histology only to study coral disease. 46% (*n* = 16) (Figure 3h) of field studies reported various ecological survey techniques that allowed for the identification of coral disease through description of gross characteristics and quantification of disease prevalence (i.e., the proportion of the community impacted). A single study used ecological surveys to investigate the presence of organisms directly associated with causing tissue loss (e.g., *Drupella*).

### 3.4 Sampling and tissue processing methodologies

**FIGURE 4:**
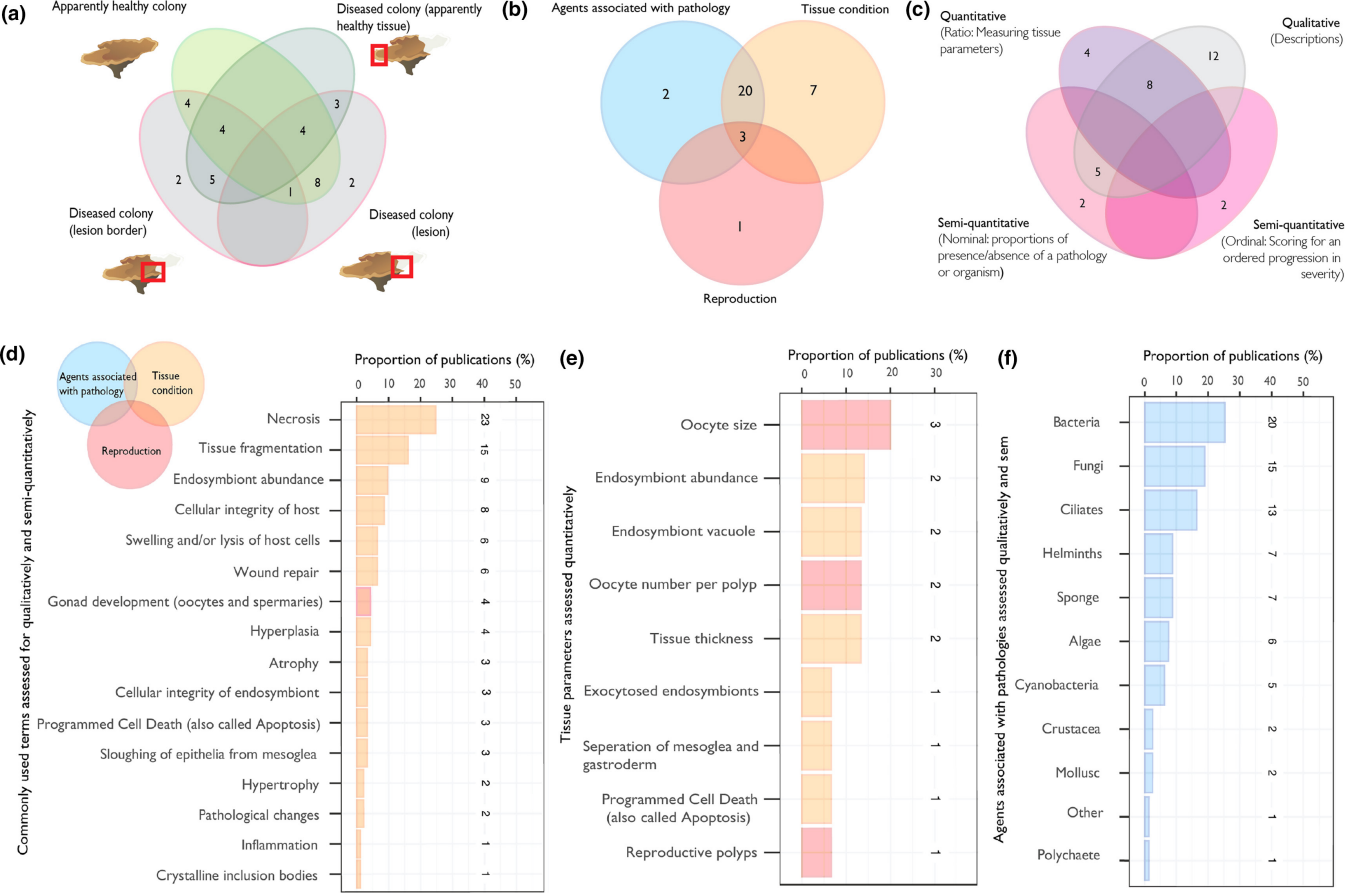
(a) Venn diagram showing the number of studies that included sampling of four different sample types: Apparently health colony (light green), apparently healthy sample from a diseased colony (dark green), sample of the lesion border of a diseased colony (pink outline), and sample of the lesion of a diseased colony (pink outline). Numbers represent counts of publications. (b) Venn diagram showing the number of studies that used histology to assess tissue condition (orange), reproduction (red) and agents associated with pathology (blue). Numbers represents counts of publications. (c) Venn diagram showing the analytical method used to assess histology: Qualitative (descriptive, grey), quantitative (numerical, purple) and two types of semi‐quantitative data formation (transformation of qualitative into quantitative data, pink) that are scoring of tissue forming nominal data (proportions of samples with presence/absence of pathologies) or organisms and semi‐quantitative scoring forming ordinal data based on ordered categories that yield a discrete value. (d) Proportion of publications that qualitatively or semi‐qualitatively made assessment of terms related to tissue condition (orange bars) and reproduction (red bars). Numbers beside bars are a count of publications. (e) Proportion of publications that used quantitative methods to make assessment of parameters related to tissue condition (orange bars) and reproduction (red). Numbers beside bars are a count of publications. (f) Proportion of publications that made assessment of agents associated with pathology. Numbers beside bars are a count of publications.

Studies conducted sampling from apparently healthy colonies with no disease signs (i.e., lesions, manifestations of disease) and sampling from colonies with active signs of disease (Figure 4a). Three types of sample were recorded as being taken from colonies with active disease signs: (1) the disease lesion, (2) the lesion border (i.e., adjacent apparently healthy tissue and tissue with disease signs) and (3) apparently healthy samples from colonies without lesions. Eight publications sampled apparently healthy colonies and lesions from colonies with active disease signs (Figure 4a). Twelve studies did not include analysis of samples from apparently healthy colonies (Figure 4a). Of these 12 studies, eight included analyses of apparently healthy samples from colonies with active disease signs (Figure 4a).

Once sampled, specimens are placed in liquid fixing agents. In total, eight fixatives were reported as being used by studies: formalin, Helly's fixative, paraformaldehyde, glutaraldehyde and seawater‐diluted Z‐Fix Concentrate (Anatech LTD, USA). The most common fixative reported was paraformaldehyde (*n* = 12) and seawater‐diluted Z‐Fix Concentrate (Anatech LTD, USA) (*n* = 15). Removal of the coral skeleton from coral samples to allow slicing of soft tissue samples occurs during a process called decalcification. Publications reported several reagents used for this process: Cal‐ex II (Fisher Scientific), ethylenediaminetetraacetic acid (EDTA), formic acid, hydrochloric acid (HCl) and a combination of HCl and EDTA. The most common reagents for decalcification were EDTA (*n* = 12) followed by formic acid (*n* = 9). A single study did not report the reagent used for decalcification as another study was referenced in place of giving methodological detail. Ten publications enrobed coral samples in agarose prior to decalcification. Once fully decalcified, specimens are processed in ethanol, xylene and paraffin washes for tissue dehydration, wax infiltration and embedding before the sections can be cut from the specimen. Thirty studies used paraffin wax for this stage, the remaining studies used resin for embedding (*n* = 3) and conducted transmission electron microscopy, in addition to light microscopy. Following embedding specimens are sectioned, and tissue sections are mounted on glass slides. In total 28 of the 33 studies reported the thickness of tissue sections cut. Tissue section thickness varied between 0.5 and 7 μm, and 5 μm was the median thickness reported. Nine of the 33 studies reported details on number of tissue sections examined per sample. Sections per sample examined varied between 1 and 10. Once mounted onto slides, tissue sections are stained with routine stains to visualise cellular structures in addition to special stains for identifying microorganisms, cellular components and structures. Twenty eight of the 33 studies reported use of haematoxylin and eosin (H & E) with 11 studies applying only H & E staining procedure to visualise tissue sections. A table of all the staining procedures reported in more than two publications visualised using light microscopy is presented in Table 3. All stains and descriptions are presented in Table S2.

**TABLE 3:** Stains for light microscopy used in two or more publications and description of what the stain is used to visualise.

Stains	What does the stain visualise and how	Number of publications
Haematoxylin and Eosin (H&E)	H&E is a routine stain that helps differentiate cell and tissue types. Nuclei are stained blue/purple, and the cytoplasm of cells are stained pink.	28
Grocott's methenamine silver	Used to visualise fungi. The fungal cell wall is stained black/brown, and the background cells will be stained green.	8
Periodic Acid Schiff's reagent (PAS)‐haematoxylin	Fungal hyphae in addition to polysaccharides and mucosubstances. Glycoproteins found in the fungal cell walls a purple/magenta colour.	5
In situ end labelling (ISEL) Programmed Cell Death assay	Used to visualise programmed cell death (apoptosis). Apoptotic nuclei (fragmented DNA) are stained brown/black, and the other nuclei are stained blue.	4
Trichrome (Mallory's, Masson's and Gomori's)	A routine stain that helps differentiate cell and tissue types. Connective tissue is stained blue/green, the cytoplasm is stained red/pink and the nuclei are stained brown/black.	4
Giemsa	The Giemsa stain can be used to look for parasites such as protozoa, fungi and Gram‐negative bacteria (e.g., Rickettsiales‐like organsisms, RLOs). Nuclei are stained dark blue, the cytoplasm is stained light blue, and parasites are stained red.	3
Phloxine B	Phloxine B is a dye typically added to H&E to enhance the red seen in H&E staining	2
Alcian blue	Mucopolysaccharides in mucus. Mucus is stained blue and surrounding tissue pink.	2
Metanil yellow	Metanil yellow is a counterstain that targets collagen in connective tissue	2
Thionin	DNA is stained green/blue and other tissue components appear red/pink.	2
Brown and Brenns	Visualises Gram‐positive and gram‐negative bacteria in tissues. Gram‐positive bacteria are stained a darker blue/purple, Gram‐negative bacteria is stained a lighter red/pink, nuclei are stained red, and the background will stain yellow.	2
Fuelgen	The DNA is stained a red/pink and the background is stained green/blue.	2
Gram stains (general)	Confirm the presence of Gram‐positive and Gram‐negative bacteria. Different types of gram stains will stain different colours, but very generally Gram‐positive bacteria is commonly stained purple, and Gram‐negative bacteria is commonly stained red/pink.	2

*Note*: A full list of stains and visualisation techniques applied in this study are found in Table S2.

### 3.5 Coral histology methodologies

Across all studies histology was used to microscopically assess coral tissue condition (integrity of the host and endosymbiont structures), reproduction and to identify agents associated with disease pathology (Figure 4b). 61% of studies (*n* = 20) assessed for tissue condition and identification of agents associated with disease pathology (Figure 4b). Seven studies used histology to assess tissue condition only, two studies focused on agents associated with tissue pathology and a single study assessed reproduction only (Figure 4b). Three studies used histology to investigate all three topics.

Publications used qualitative (descriptive), quantitative (ratio data) and semi‐quantitative (transformation of qualitative into quantitative data that is nominal or ordinal) analysis methods (Figure 4c). Two types of semi‐quantitative data formation were used: (1) ordinal scoring of tissue based on ordered categories of severity that yield a discrete value and (2) nominal data that forms proportions of samples (i.e., prevalence) with the presence/absence of pathologies or organisms (Figure 4c). Two publications used semi‐quantitative scores applied to samples to assess group differences. Twelve studies presented qualitative analysis of tissue sections, whilst other studies combined qualitative observations with either semi‐quantitative measures (prevalence, *n* = 5) or quantitative measures (*n* = 8) (Figure 4c). Four studies used purely quantitative assessment of histological sections. Publications that qualitatively or semi‐qualitatively assessed tissue used terms to describe tissue condition and reproductive state (Figure 4d).

### 3.6 Use of terminology for describing histopathological observations

Of the 30 studies that reported on tissue conditions, we found 16 terms that were used qualitatively and quantitatively to describe histological features (Figure 4d). Publications most consistently made statements associated with the following terms to describe tissue condition: necrosis (*n* = 23), tissue fragmentation (*n* = 15), endosymbiont abundance (*n* = 9), cellular integrity of the host (*n* = 8), swelling and/or lysis of host cells (*n* = 6) and wound repair (*n* = 6) (Figure 4d). Four publications made statements about stages of gonad development (Figure 4d). See Table 5 for the definition of these terms to describe tissue condition and references for examples of studies that used these terms. Nine tissue parameters were assessed quantitatively in publications (Figure 4e). Quantitative measures specific to reproduction were measures of oocyte size (i.e., volume) (*n* = 3), oocyte numbers per polyp (*n* = 2) and the number of reproductive polyps in a tissue section (*n* = 1) (Figure 4e). Other quantitative measures were associated with tissue condition and include counts of endosymbiont abundance (*n* = 2), endosymbiont vacuole ratios (vacuolisation) (*n* = 2), tissue thickness (*n* = 2), counts of exocytosed endosymbionts (*n* = 1), measurement of the separation distance between mesoglea and gastrodermis (*n* = 1) and counts of apoptotic nuclei (*n* = 1) (Figure 4e). Twenty five total publications examined tissue sections for 11 possible disease‐associated agents (Figure 4f). Bacteria were the most cited organisms found in examined tissue sections (*n* = 20). Publications also examined tissue for fungi (*n* = 15) and ciliates (*n* = 13), helminths (*n* = 7), sponges (*n* = 7), algae (*n* = 6), cyanobacteria (*n* = 5), crustacea (*n* = 2), molluscs (*n* = 1), polychaetes (*n* = 1) and other (*n* = 1) which refers to invasive gastrovascular multicellular structures (IGMS see Work et al., 2012). See Table 6 for morphological characteristics associated with each of these agents and references to publications for examples.

### 3.7 Critical appraisal of coral histology methodologies

Appraisal of resource availability showed that 94% (*n* = 31) of publications specified a lead contact. Raw data were released in 36% (*n* = 12) of studies and 12% (*n* = 4) of studies did not release raw data but specified release on contact with the authors. None of the publications included within this review used code for statistical analysis or data visualisation (Table 4). In total, 18% (*n* = 6) of publications cited a separate publication instead of providing complete details of the methodology used (Table 4). Study species were listed to species level in 91% (*n* = 30) of studies (Table 4). Of those studied that used aquaria facilities (*n* = 6), four provided details of aquaria maintenance; however, none of the four studies provided information necessary to replicate culture conditions, whereas two publications provided all details for aquaria maintenance (Table 4). Information on time of sampling was most often provided as a month of sample collection (36%, *n* = 12), a single study stated year of collection, and others reported sampling over a specific time period (27%, *n* = 9). In total, 18% (*n* = 6) of publications did not specify a sampling time point and only four provided the exact date of sampling (Table 4). No studies reported time of day for collection of samples. Longitude and latitude of the sampling/study locations were provided in 48% (*n* = 16) of studies 39% (*n* = 15) reported a reef location name (Table 4). Three publications only reported a country of sampling, and one publication did not clearly state a site of sampling (Table 4).

**TABLE 4:** Critical appraisal of methodology detailed as the number of publications out of all total publications included within the review.

Resource availability	Lead contact: Details specified?	Yes = 31/33 No = 2/33
	Are the raw data released with the study?	Yes = 12/33 No = 17/33 Contact author = 4/33
	If code is used, is it released with the study?	No code specified in any studies
Method details	Are other paper(s) cited in replacement of providing adequate details of the procedure applied?	Yes = 6/33 No = 27/33
	Are the species studied listed?	Yes = 30/33 No (genus level only) = 3/33
	If experimental, are full details on aquaria maintenance provided?	NA (no aquaria used) = 27/33 Some details but not enough to repeat the experiment = 4/33 All details have been specified = 2/33
	Are information on timing of sampling provided?	Date (d//m/y) = 4/33 Month = 12/33 Year = 1/33 Time frame = 9/33 Not specified = 6/33
	Are information on sampling location provided?	GPS coordinates = 16/33 Reefs specified = 13/33 Country only = 3/33 No location = 1/33
	Are sample sizes provided?	
	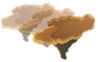 Colonies per sample group	Not specified = 2/33 Different per taxa, disease type or sample group = 17/33 Consistent sample sizes = 13/33
	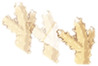 Fragments per sample group	Not specified = 2/33 Specified = 30/33 Range given = 1/33
	Is masking applied during evaluation of tissue?	Yes = 1/33 Not specified = 32/33
	Is randomisation applied during evaluation of tissue?	Yes = 1/33 Not specified = 32/33
	Are definitions of tissue assessment terms provided?	Yes = 11/33 No = 19/33 For some = 3/33
	Is the method for how tissue were assessed provided? (e.g., the tissue area assessed per sample, the tissue layers assessed per sample)	Yes = 8/33 No = 25/33
Quantification and statistical analysis	If statistics are used, are all test completed stated?	NA = 11/33 Yes = 22/33
	If statistics are used, is explanation for how significance is assessed defined?	Yes = 4/22 No = 18/22

When reporting sample sizes 51% (*n* = 17) of publications were unclear, or reported unequal sample sizes for each type of sample group (e.g., diseased and apparently healthy), and for different taxa studied for each disease (Table 4). Biological replicates at the level of a colony accounts for variation within individuals but reporting on biological replicates was also inconsistent. The number of biological replicates per sample group (i.e., fragments per colony) was reported by 91% (*n* = 30) of publications and one publication specified a range (1–2 fragments per colony) (Table 4), while 28 studies sampled one fragment of each sample type per replicate colony, one study took two sample types per replicate, and one study sampled four sample types (replicates) per colony. Two studies in total reported on using masking or randomisation during histology methods (Table 4). In 56% (*n* = 19) of studies definitions of the terms or categories used to assess tissue were not provided (Table 4). Seven studies in total provided reproducible methodology for how tissue was assessed, six of which were quantitative and two were semi‐quantitative. Each of these studies took different approaches to assessment of tissue. This included statements of units of cell types or tissues assessed per sample. Two studies divided analysis based on tissue type: surface body wall and basal body wall. Of the 22 studies that used statistical tests, only four reported an explanation for how statistical significance was determined.

## 4 DISCUSSION

Ongoing climate change, increasing anthropogenic impacts and growing reports of coral disease within the scientific literature highlights the urgency for developing standardised, comparative approaches for diagnosis of coral disease using microscopy. Histology can provide key insights for elucidating disease causation, disease impacts on coral colonies and therefore assessing management options during outbreaks. However, standardised reporting guidelines and ongoing, transparent means to compare research has not yet been developed for the field of coral disease research. In this study, we provided:

1. A systematic review protocol for repeatable identification of research into white diseases affecting hard coral, and
2. A systematic review of literature identified from 1984 to April 2023.

From our systematic review we propose:

1. Reporting standards for microscopic studies of white diseases to support future comparative analysis including suggested reporting information within methods for inclusion in future systematic reviews, and
2. The need for living reviews of coral disease literature overall, and specific diseases including the assessment of diseases in hard corals using histology.

Together these are required to improve understanding of coral diseases, establish trends in disease events and disease literature, in addition to allowing comparative assessment of disease outbreaks overtime. We further argue that doing so provides support for researchers on coral reefs observing disease outbreaks to plan and implement studies investigating disease causation and impacts, access information of gross and microscopic disease signs and coral pathologies and ultimately increase the body of work that uses histology to assess and compare coral health.

### 4.1 Overall findings of systematic review

The application of histological methods to understand white diseases in hard corals has yielded compelling insights into diagnosis and disease impacts on coral function including reproduction (Ainsworth, Kramasky‐Winter, et al., 2007; Andersen et al., 2010; Gignoux‐Wolfsohn et al., 2020; Landsberg et al., 2020; Sudek, Aeby, et al., 2012; Work & Aeby, 2006). However, we show uptake of histology for the study of white disease has been slow within the field. The earliest publication identified applied histology to assess white disease in 1984 (Peters, 1984). White band disease was examined in *Acropora* colonies collected from St Croix and Puerto Rico, Caribbean and 10 stains were applied to examine reproduction, coral‐algal interactions, bacteria, cell structure and microparasites (Peters, 1984). The author concluded that histology allowed accurate assessment of the presence of microorganisms and the study correlated tissue state, microparasite infestations with apparent disease signs (Peters, 1984). However, despite the conclusions reached by Peters (1984) the next publication our systematic review methodology returned was 17 years later, published in 2002 (Bythell et al., 2002). In 2014, a coral disease literature review also highlighted the disparity in methodology used in coral disease studies (Work & Meteyer, 2014) reporting that prior to 2013 only 12% of the total publications of coral disease applied standard histology and light microscopy to investigate microscopic pathology, compared with 65% of reports undertaking ecological surveys to report disease (Work & Meteyer, 2014). Note that in this study, we did not collate literature on white diseases using methods (such as ecological surveys) without applying histology methods. Following 2002, our protocol returned 17 studies from 2002 to 2013 and then 18 studies in the 10 years following. While there is an increase in the use of histology in the study of coral disease over the last 2 decades, these results suggest that further work is needed to encourage the uptake of histology methods in the coral disease field.

We find women contribute substantially to leadership (herein defined as first, second and last author roles) within this field of coral reef science with 28.8% of authors identifying (in open access online resources) as female and 42.5% as male. In terms of last author role we found 18% female and 56% male, compared with the study of Ahmadia et al. (2021) who reported last authorship across the coral science field is dominated by men (80% in 2018). Similarly, as seen across the coral reef research field (Ahmadia et al., 2021) in this study author representation from high income, developed countries dominated the leadership of research effort to date with some evidence of collaboration between developing and developed nations. Ahmadia et al. (2021) highlight there is an ongoing disconnect between geographic origins of scientific knowledge for coral research and the locations of the world's coral reefs. Coral histology requires specialised equipment, and research coming from a limited number of dedicated research laboratories is to be expected. As identified by several studies molecular tools for assessment of coral health (e.g., microbiome or other ‘omics’; Traylor‐Knowles et al., 2022) can be inaccessible to reef managers due to the need for specialist equipment in addition to sample processing, high cost analysis and specialist data analysis (Donner & Potere, 2007). Histology can be an accessible method for the assessment of coral health assessment due to ease of sample collection, storage and analysis if methods are accessible and openly available. Our results emphasise the need for continued efforts for interdisciplinary collaboration, and knowledge‐sharing to facilitate the use of histology in coral disease research. This is particularly evident when considering outbreaks of coral disease, of which reef managers are most likely to be the first observers of outbreaks. Early identification of disease signs and sampling during disease emergence can enable identification of potential causative agents of the disease to be more easily differentiated from secondary colonisers (Lesser et al., 2007).

Across the studies identified through the systematic literature search, 26 separate stains were used to visualise various structures and organisms. Eleven studies reached conclusions applying a single routine stain, haematoxylin and eosin. Studies included in this review also used histology to assess reproduction and four studies in total made assessment of reproductive parameters in diseased tissue. Different research aims were also stated across the studies including assessment of tissue condition, reproduction and identification of other non‐coral organisms. Our review finds that for the studies undertaken to date, white diseases of hard corals are associated with a wide array of different bacterial and ciliate associates, as well as numerous other microorganisms such as fungi, viruses and parasites (Luna et al., 2010; Sweet & Bythell, 2015; Work & Aeby, 2011). Additionally, studies identified endolithic algae (Fine et al., 2006), fungi (Howells et al., 2020), bacterial structures (Sere et al., 2013) and ciliates (Smith et al., 2020) as possible secondary invaders of diseased tissue.

### 4.2 Recommendations for standardising reporting of coral histology

#### 4.2.1 Reporting quality

**FIGURE 5:**
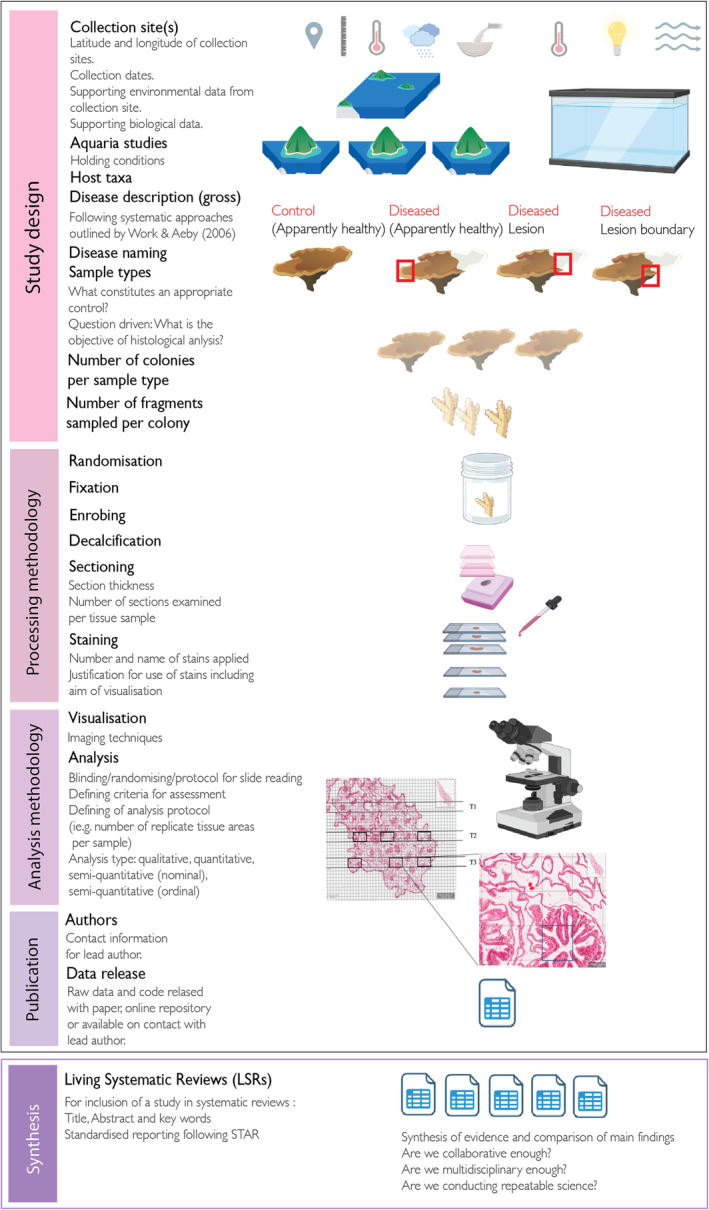
Recommendations for reporting in coral disease studies using histology across study design, processing, analysis and publication. Icons free to reuse and are sourced from BioRender (https://app.biorender.com/) and from the University of Maryland media library (https://ian.umces.edu/).

In this study we used the STAR (Structure, Transparent, Accessible Reporting) Methods protocol outlined by Cell Journal (Marcus, 2016) in addition to work by Gibson‐Corley et al. (2013) presenting best practice for scoring histopathological tissues to develop criteria in which to appraise the quality of methods reporting in each of the studies. We find quality of reporting across sampling, processing and analysis of tissue sections has not consistently met standards. Poor usability, defined as a difficulty in evaluating what was done by a study, leads to an inability to reuse or repeat methodology, making cross comparisons between studies and incorporating evidence into systematic reviews and meta‐analysis difficult (Munafò et al., 2017). Reproducible research in contrast results in faster methodological development and innovation because research is accessible to more scientists (Alston & Rick, 2021). Recent reviews of biomedical and public health, including slide‐based research, found that articles generally lacked rigour and transparency in one or more fundamental areas needed for reproducing studies (Freedman & Inglese, 2014; Naudet et al., 2018; Wallach et al., 2018). Because practices have been found to be inadequate, various groups have aimed to address transparency through development of reporting guidelines (Chiriboga et al., 2022; Kenall et al., 2015; Marcus, 2016). As highlighted in the recent study by Hawthorn et al. (2023), key to moving forward the coral disease field is more studies of coral histology of both natural and experimental disease, as well as healthy coral samples from a variety of environments. On reviewing reporting practices in methodology for coral disease histology alongside STAR protocols (Marcus, 2016) and best practice for histological tissue scoring (Gibson‐Corley et al., 2013), we are able to recommend guidelines for reporting of study design, sample processing, analysis and publication for future studies of coral disease (Figure 5).

#### 4.2.2 Study design

A critical step in reproducibility of histological methods is the experimental design which can reduce biases in the analysis of tissue (Gibson‐Corley et al., 2012). Key considerations to address include appropriate description of sample collection sites, including latitude and longitudes, and dates and times of sample collection and for coral reef research collection of environmental metadata where possible (such as temperature conditions, water quality metrics and site descriptions to aid in the replication and comparative analysis of studies) (Figure 5) (see also online resources for Coral Disease & Health Consortium; https://cdhc.noaa.gov/). We find that a single experimental study did not provide any reference for a collection country or reef site, with corals reported as obtained from aquaria facilities but source location not stated in the methodology. For experimental studies reporting of culture conditions is also valuable, including details of acclimation periods following collection, aquaria temperature regimes, light conditions and if possible flow rates (see Grottoli et al., 2021 for other aquaria recommendations). Although it is well recognised that environmental factors like temperature, depth, turbidity and flow (Page et al., 2019) can all affect the biology of the coral holobiont, we found that 49% of articles did not provide metadata to support contextualising the environment of sourced samples. In addition to depth, temperature and flow conditions, contextual information on drivers of coral health and disease like pollution levels and rainfall from sourced populations is also important to understanding coral biology in diseased as well as apparently healthy individuals (Downs et al., 2009; Edge et al., 2013; Haapkylä et al., 2011; Page et al., 2023). For example corals have been found to respond differently to environmental stressors within different reef habitats (Ainsworth et al., 2021). Therefore, including information on ‘reef habitat’ (e.g., reef slope, sheltered reef slope and reef flat) following classification by the Allen Coral Atlas (Kennedy et al., 2020) can allow researchers to later consider environment in comparative studies.

#### 4.2.3 Host taxa, gross disease descriptions and nomenclature

An important consideration for coral disease research is the challenge of taxa identification particularly in understudied regions where taxonomic classification can be uncertain, classifications remain unresolved and taxonomic sources/records are unclear. Online resources such as World Register of Marine Species (www.marinespecies.org), the World List of Scleractinia (http://www.marinespecies.org/scleractinia) and reporting databases such as redmap.org (Pecl et al., 2014) and iNaturalist (Matheson, 2014) can be useful for access to species observations in addition to museum resources and specialists. Work and Aeby (2006) (and see https://cdhc.noaa.gov/) provide recommendations for standardised and systematic observations of disease signs to aid in halting the proliferation of distinct names for similar disease signs, with often a lack of defined and distinguishable aetiologies. For example, tissue loss diseases with no clear distinguishable features (i.e., a brown or black band) are recommended to be referred to as ‘white syndrome’ (Bourne et al., 2015) and diseases named in reference to the taxa they impact (e.g., ‘*Acropora* white syndrome’) as it is understood that diseases that present similarly in different taxa can have different underlying drivers and therefore should be treated as separate diseases (Aeby et al., 2011; Rogers, 2010; Sussman et al., 2008).

#### 4.2.4 Controls: Apparently healthy individuals

Critically selection of the appropriate controls and calculation of sufficient sample sizes needed for statistical tests are also widely recommended for histological research to discern abnormal pathology (Meyerholz & Beck, 2018a). Here we find 12 studies of hard coral white diseases did not include samples of apparently healthy colonies (i.e., not affected by the described disease). Including ‘healthy’ controls within studies can be a challenge during outbreaks as visually healthy colonies may at the cellular level be stressed (Ainsworth et al., 2008). However consistent terminology to describe tissue pathologies and metadata on abiotic pathogens can assist in differentiating disease states. To overcome this, a single study used reference samples taken from apparently healthy colonies years before the study period and disease outbreak as a comparison to assess tissues, highlighting the value of continuous site assessment where possible (Miller et al., 2014).

#### 4.2.5 Histological tissue scored

We also found that few studies clearly specified the method for how sample tissue was scored. Ideally, the scoring procedure should be clearly stated and clear language should be used when defining terms, or categories of changes used to score samples (Gibson‐Corley et al., 2013; Meyerholz & Beck, 2018a, 2018b). We find that there are several types of tissue scoring that have been applied by authors to assess disease pathology and group differences between sample types. Twenty‐five publications out of the 33 reviewed in total provided some qualitative description of tissue, with 12 of these providing qualitative descriptions only. Qualitative descriptions can be useful in providing initial diagnostic and descriptive changes observed between samples, but there are limitations for more rigorous insight into group comparisons (Table 7). Here, the formation of quantitative or semi‐quantitative data from tissue is recommended to aid diagnosis (Weber, 1988) and/or answer other research aims following methods outlined in (Gibson‐Corley et al., 2013; Holland & Holland, 2011) (Table 7). Specifically, the formation of semi‐quantitative ordinal data is a commonly used practice in medical pathology (Meyerholz & Beck, 2018b). We find that there are two studies that use semi‐quantitative approaches to disease. These studies assigned tissue a score from 0 to 5 based on severity (0 = excellent, 5 = very poor) for endosymbiont condition and abundance and six parameters of polyp health (both cell and tissue) and bacteria (Gignoux‐Wolfsohn et al., 2020; Miller et al., 2014).

The foundational concepts for assessment of diseased tissues are similar across scientific areas (Chiriboga et al., 2022; Gibson‐Corley et al., 2013; Meyerholz & Beck, 2018b; Wolf et al., 2015) and include principles associated with bias control in tissue evaluation (Chiriboga et al., 2022; Meyerholz & Beck, 2018a). For example, bias can be reduced through steps at sample processing stage that involve following a set protocol for slide assessment (Gibson‐Corley et al., 2013; Holland & Holland, 2011). These include complete randomisation through labelling individual samples without reference to treatment group (e.g., 1, 2, 3, 4, 5…) or protocols like the pair‐contrast method where treatment and control (i.e., diseased and apparently healthy individuals) are randomly paired and the most affected sample from each pair is noted (Gibson‐Corley et al., 2013; Holland & Holland, 2011), Ideally, slides should be examined three times with full (or partial, e.g., 10% of slides) peer‐review of slide scoring. Gibson‐Corley et al. (2013) also suggest limiting ‘diagnostic drift’ (where assignment of scores vary slightly in consistency through the scoring process) by slides being examined over a reasonable amount of time. There are also several methods for validating scoring systems including observer repeatability and tissue pathobiology. Details of these can be found in Gibson‐Corley et al. (2013) in addition to (Cross, 1996; Germolec et al., 2004; Landis & Koch, 1977). At the slide‐reading stage authors should also be sure to provide information on the number of sections examined per sample, and the number of sections examined per stain, per sample. This biological replication is important for accounting for variation within a single sample.

#### 4.2.6 Nomenclature for microscopic assessment

Inconsistencies in nomenclature has been identified as an ongoing issue in coral disease research with various reports of disease signs referring to the same disease (e.g., ‘black band disease’ and ‘black aggressive band’) (Moriarty et al., 2020). The use of clear diagnostic terminology is strongly recommended when assessing tissue, as the use of non‐definable criteria limits the conclusions that can be drawn from analysis (Gibson‐Corley et al., 2013; Meyerholz & Beck, 2018b). We find only 11 out of the total 33 papers included in this study were ‘repeatable’ as they provided definitions of terms used to describe coral tissue. In this study we highlight the most widely used terms to describe coral tissue pathologies: necrosis, tissue fragmentation, hyperplasia, inflammation, suspect wound repair, atrophy and hypertrophy (Table 5). We also provide details of the morphological characteristics of agents associated with coral diseases identified by publications (Table 6). In these tables we provide citations to publications included in the review where images of each pathology and associated organisms have been provided. These lists are not exhaustive and we suggest that authors consult both peer‐reviewed literature for descriptions and definitions of histological terms and anatomical structures (i.e., see Hawthorn et al., 2023; LaDouceur, 2021; Woodley et al., 2016 in addition to online resources for coral disease and histology such as the Coral Disease & Health Consortium [https://cdhc.noaa.gov/]).

**TABLE 5:** Morphological description and characters of general terms used to describe conditions of coral tissue identifiable under routine staining (H & E).

Term	Morphological description and characteristics under routine staining (i.e., H & E)	Reference for publication(s) included within this review with images of examples
Necrosis	Necrosis is the accidental or passive death of cells (Syntichaki & Tavernarakis, 2002) characterised by cellular swelling and lysis leading to leaking of intracellular contents. The outline of individual cells will be indistinct, sometimes forming a focus of granular material. One commonly cited sign of necrosis is pyknosis, the contraction of the nucleus to a deep staining blue irregular or round mass.	
Tissue fragmentation	Variable sized clumps of intact cells (Work et al., 2016).	
Hyperplasia	Widespread proliferation of cell types (gastrodermis, epidermis, mesoglea and calicodermis) alongside reduced formation or lack of mesenterial filaments or polyp structures.	Work et al. (2016)
Inflammation	A process aimed at destroying, diluting or walling off infectious agents, characterised by infiltration of tissues with larger‐than‐normal amounts of mesogleal cells.	Aeby, Shore, et al. (2021) and Work et al. (2016)
Suspect wound repair	Fragmented tissues with evidence of regeneration of undifferentiated epidermal tissue (e.g., no clear gastrodermis and epithelial layers are present).	Work and Aeby (2011)
Atrophy	Generalised shrinking of the epidermis or gastrodermis.	Work et al. (2012)
Hypertrophy	The increase in the volume of an organ or tissue due to the enlargement of its component cells.	Thome et al. (2021)

*Note*: For further explanation of tissue pathologies, see Hawthorn et al. (2023), LaDouceur (2021) and Woodley et al. (2016).

**TABLE 6:** Morphological characteristics and references for agents associated with coral disease.

Organism	Morphological description and characteristics under routine staining (i.e., H & E)	Reference for publication(s) included within this review with images of examples
Helminths	Non‐segmented, worm shaped (vermiform) metazoan with or without gut	Sudek, Work, et al. (2012) and Work et al. (2012)
Sponge	Metazoa with connective tissue matrix containing spicules and choanocytes. There may be presence of endosymbionts.	Work and Aeby (2011) and Work et al. (2012)
Algae	Metazoa with cell walls.	Work et al. (2012)
Fungi	Elongate branching, irregular filamentous structures with or without septa.	Howells et al. (2020) and Work and Aeby (2011)
Crustacea	Metazoa with gut, muscle, reserve inclusion cells, cuticle, hepatopancreas and segmented appendages.	Work et al. (2012)
Ciliates	Allantoid (round, slightly elongated) unicellular ciliate covered organisms. Can be found invading gastrovascular canal associated with necrosis. Ciliates can be present with ingested endosymbionts.	Work and Aeby (2011) and Work and Rameyer (2005)
Cyanobacteria	Parallel walled, filamentous striated structures.	Fine et al. (2006) and Work et al. (2012)
Bacteria	Diverse structures that vary in size including coccoid like and coccobacilloid like, in addition to larger bacterial aggregates.	Landsberg et al. (2020) and Sudek, Work, et al. (2012)
Mollusc	Large metazoan with gills and striated muscle. Eyes and radula sometimes visible.	Work et al. (2012)

**TABLE 7:** Types of semi‐quantitative and quantitative data that can be obtained from histological tissue.

Type	Definition	Considerations
Semi‐quantitative, Nominal	Samples assigned to a category without references to severity. Examples include binary presence or absence, or categorical assignment of lesions (e.g., necrotic)	This approach can record the number of samples affected by a specific pathology (i.e., prevalence) as a proportion. It gives no information on the extent of severity of a pathology in samples.
Semi‐quantitative, Ordinal	Samples assigned to a category showing an ordered progression in severity.	A summary score can be produced for each sample. If multiple tissue fields are assessed per sample a mean score can be assigned. For ordinal data, the median is the most appropriate measure of central tendency for groups. Scores could be developed for each tissue pathology of interest (e.g., host condition, endosymbiont condition and reproductive state) or a single overarching scoring system could be applied. The latter system can lead to difficulty in scoring when pathologies do not co‐occur in regular patterns. Consideration of the number of scoring categories is important, too little can lead to reduced sensitivity to detect biological differences and a larger number can cause difficulty in score assignment due to less obvious differences between categories. It has been suggested that 4–5 scoring levels is an optimal range to maximise detection and repeatability. Semi‐quantitative scoring provides approximate relative changes in tissue and can be less time intensive than quantitative measures from tissue while still providing information on group difference.
Quantitative, Ratio	Data are quantified on a scale with a true zero value.	Samples can be compared through differences or multiplication/division. For coral tissue, this method could be time intensive as multiple replicate measurements or counts will need to be assessed per tissue section to account for inherent heterogeneity in coral samples.

*Note*: Adapted from Gibson‐Corley et al. (2013).

#### 4.2.7 Reporting standards

STAR protocols for methods reporting (Marcus, 2016), best practices for reproducible research (Gundersen, 2021; Munafò et al., 2017), and increasing knowledge‐sharing through providing author/data contact information and accessible raw data are all now becoming norms across research disciplines. A field‐wide development of reporting standards specific to coral disease reports would also aid in comparative and collaborative research. Towards tackling systemic inequalities present in STEMM fields including coral reef science (Ahmadia et al., 2021; Runnels et al., 2014; Tricco et al., 2023), increasing representation and collaboration within the field, the use of evidence syntheses are important ways forward in building the research field as the coral reef crisis continues globally.

## 5 CONCLUSIONS

As coral diseases and coral bleaching have become an increasing threat to coral reefs in the last few decades, knowledge of the microscopic structure, composition of coral tissue and what drives poor coral health has accumulated but there remain many crucial knowledge gaps and inconsistencies in the field. Histology to look at coral tissues is a flexible tool that can help assess coral holobiont function and aid in disease diagnostics. In this review, we use a systematic protocol to map publications that have used histology to study white diseases in corals and review the methodological design and procedures used. The results of this study highlight advantages to histology; however, we find that studies tend to lack detailed methodological information reducing the useability of research. Using this body of the literature, we present the most widely used tissue terminology in addition to recommendations for methodology and reporting of future histological studies, with the aim of increasing accessibility, comparability and encouraging uptake of histology in future studies of coral disease.

## AUTHOR CONTRIBUTIONS

**C. E. Page:** Conceptualization (lead); data curation (lead); formal analysis (lead); investigation (lead); methodology (lead); visualization (lead); writing – original draft (lead); writing – review and editing (lead). **E. Anderson:** Data curation (supporting); investigation (supporting); methodology (supporting); writing – original draft (supporting). **T. D. Ainsworth:** Conceptualization (equal); funding acquisition (equal); supervision (lead); writing – review and editing (equal).

## CONFLICT OF INTEREST STATEMENT

The authors declare no conflicts of interest.

### OPEN RESEARCH BADGES

This article has earned a Preregistered Research Designs badge for having a preregistered research design, available at https://github.com/CharlotteEPage/Histology_methods_systematic_review.git and https://doi.org/10.5061/dryad.mkkwh7183.

## Supporting information


Data S1

## Data Availability

Raw data collected during data extraction of the systematic review, and code used for analysis and visualisation is available for download at https://github.com/CharlotteEPage/Histology_methods_systematic_review.git and https://doi.org/10.5061/dryad.mkkwh7183.
