# Supplementary material for: Building living systematic reviews and reporting standards for comparative microscopic analysis of white diseases in hard corals
Source: Ecol Evol. 2024 Jul 4;14(7):e11616. doi: 10.1002/ece3.11616 (PMC11224507; doi:10.1002/ece3.11616)
Supplement: Supplementary file 1 — Data S1 [file ECE3-14-e11616-s001.pdf]

Table S1. Critical appraisal of methodology adapted using STAR Method (1) and best practice for scoring of histopathological samples (2)

Resource availability	Lead contact: are the lead contact's details specified?
	Are the raw data released with the study?
	If code is used, is it released with the study?
Method details	Is another paper cited in replacement of providing adequate details of the procedure applied?
	Are the species studied listed?
	If experimental are full details on aquaria maintenance provided?
	Is information on timing of sampling provided?
	Is information on sampling location provided?
	Are sample sizes provided?
	Is masking or blinding applied to the histoslides during evaluation of tissue?
	Is randomisation applied during evaluation of tissue?
	Are repeatable definitions of tissue assessment provided?
	Are repeatable definitions of how tissue was assessed provided? (e.g. the tissue area assessed per sample, the tissue layers assessed per sample)
Quantification and statistical analysis	If statistics were used, are all tests completed stated?
	If statistics were used, are explanations for how significance was assessed provided?

Table S2. List of stains and visualisation methods used in reviewed publications and description of what is visualised with the stain.

**Stain**	**What does the stain visualise?**	**What does it look like visually?**	**Reference**
Hematoxylin and eosin (H&E)	H&E is a routine stain that helps differentiate cell and tissue types.	Nuclei are stained blue/purple, and the cytoplasm of cells are stained pink.	(e.g. see (3–16))

Heidenhain’s aniline blue	Connective tissue.	Collagen is stained blue, chromatin orange/red and the cytoplasm a pink/red.	(9)
Modified Movat’s Pentachrome	Connective tissue	Nuclei blue/black, collagen yellow, mucin bright blue and fibrin (nematocysts) bright red.	(9)
Periodic Acid Schiff (PAS)-hematoxylin	Fungal hyphae in addition to polysaccharides and mucosubstances	Glycoproteins found in the fungal cell walls a purple/magenta colour.	(5,7,9,17)
Feulgen	DNA	The DNA is stained a red/pink and the background is stained green/blue.	(7,9)
Brown & Brenn's	Visualises gram-positive and gram-negative bacteria in tissues	Gram-positive bacteria is stained a darker blue/purple, gram-negative bacteria is stained a lighter red/pink, nuclei are stained red and the background will stain yellow.	(7,9)

Grocott's methenamine silver	Fungi	The fungal cell wall is stained black/brown, and the background cells will be stained green.	(7,9,14–17)
Mallory's trichrome	Connective tissue	The nuclei are stained brown/blue, the cytoplasm is red/pink and the collagen is blue.	(11,18)
Gomori's trichrome	Connective tissue as a more simplified version of Mallory's trichrome	The nuclei are stained red/purple, the cytoplasm is red/pink and the collagen is green.	(10,19)
Masson's trichrome	Collagen in connective tissue	Collagen is stained blue/green, the cytoplasm is stained red/pink and the nuclei are stained brown/black.	(13)
Phloxine B	Phloxine B is a dye typically added to eosin to enhance protein staining.	Enhances red seen in H&E staining.	(12,20)
Giemsa	The Giemsa stain can be used to look for parasites such as protozoa, fungi and bacteria (e.g. RLOs)	Gram-negative bacteria will stain dark blue, rickettsia-like organisms purple-red, mucus may	(6,8,19)

		be stained various shades of blue and proteins in intracellular vesicles and spirocysts will stain pink.	
May-Grünwald Giemsa	Stain combines the May-Grünwald stain and the Giesma stain. Used to visualise protozoa.	Nuclei are stained blue/purple, cytoplasm is a very light pink and foreign bodies stain dark purple.	(7)
Toluidine blue	Toluidine blue is considered a routine stain for DNA	A basic dye that stains nucleic acids blue and polysaccharides purple. It often binds to all proteins.	(21)
Gram stains (general)	Confirm the presence of gram-positive and gram-negative bacteria.	Different types of gram stains will stain different colours, but very generally gram-positive bacteria are commonly stained purple and gram-negative bacteria are commonly stained red/pink.	(11,14)

Taylor's Gram stains	Gram stain used to decipher gram-positive and gram-negative bacteria	Gram-positive bacteria stain blue/black and gram-negative bacteria stain bright red.	(16)
Metanil yellow	Metanil yellow is a counterstain that targets collagen in connective tissue	Collagen is stained yellow.	(5,7)
Fontana-Masson (FM) silver	Melanin	Melanin is stained brown/black and cytoplasm pink.	(7)
Macchiavello	Identify two groups of gram-negative bacteria: chlamydia and rickettsia	Cytoplasm is stained a grey/blue colour, nuclei purple, and bacteria magenta.	(7)
Methyl green pyronin	Differentiates between DNA and RNA	DNA in the nuclei is stained green/blue and RNA red.	(7)

Perl’s Prussian blue	Stains for iron	Iron appears as blue/purple deposits	(7)
Thionin	DNA	DNA is stained green/blue and other tissue components appear red/pink.	(5,7)
In situ end labelling (ISEL) Programmed Cell Death assay	Apoptosis	Apoptotic nuclei (fragmented DNA) are stained brown/black and the other nuclei are stained blue.	(4,10,21,22)
Alcian blue	Mucopolysaccharides in mucus.	Mucus is stained blue and surrounding tissue pink.	(7)
Fluorescence in situ hybridisation (FISH)	Used to visualise the distribution and location of a specific bacteria.<br><br>Common probes include eubacterial in an equimolar mix (EUBMIX)	Bacterial detection visualised by fluorescence (e.g., CY3 red). Surrounding coral tissue will have autofluorescence.	(10,19–21)
Nigrosin	Necrosis	Necrotic cells stain black/brown.	(21) 3/06/2024 4:43:00 AM

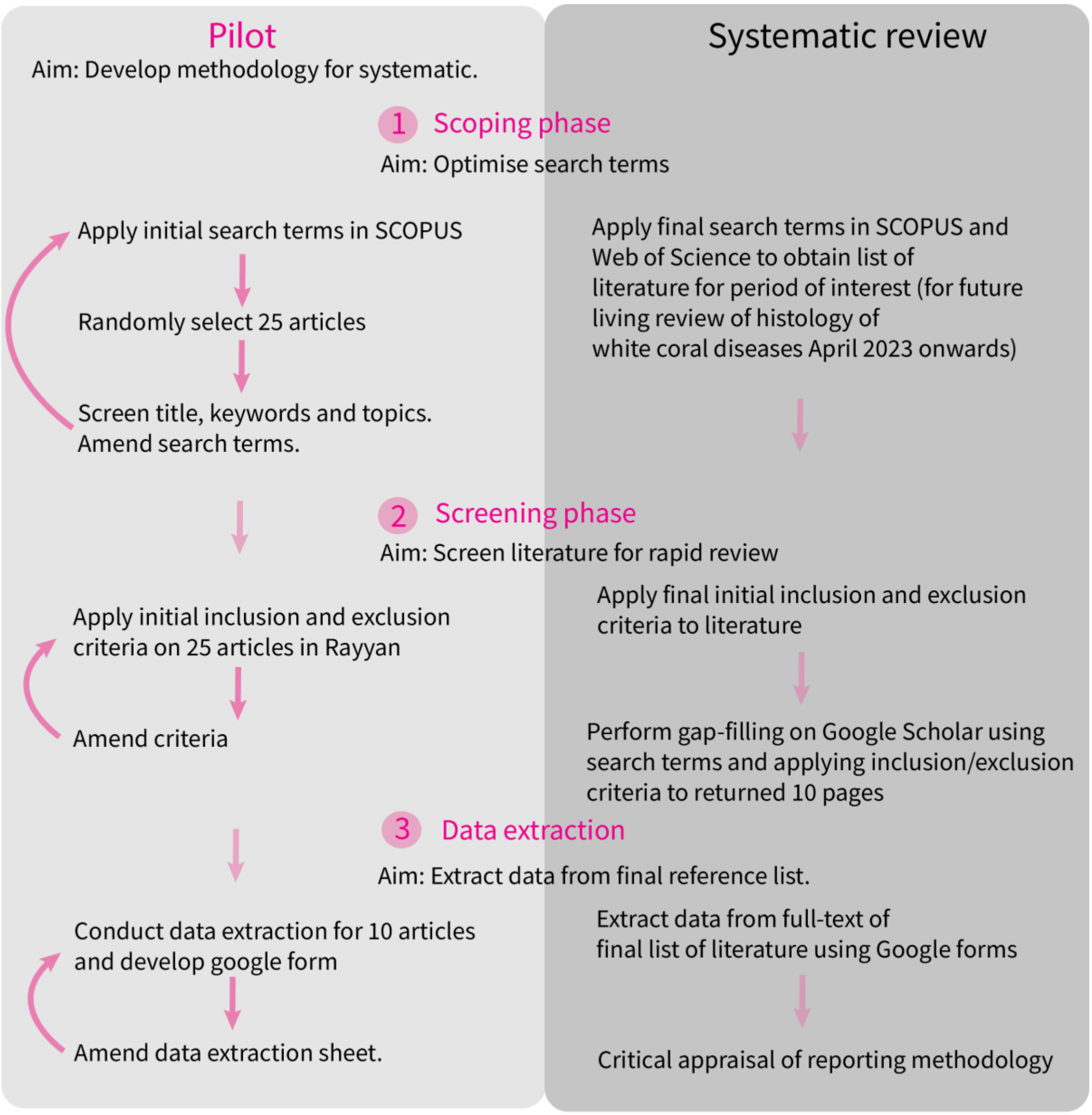

Figure S1. Conceptual diagram showing development of systematic protocol procedures, including the iterative development pilot phases, and the protocol for the final systematic review of the literature.

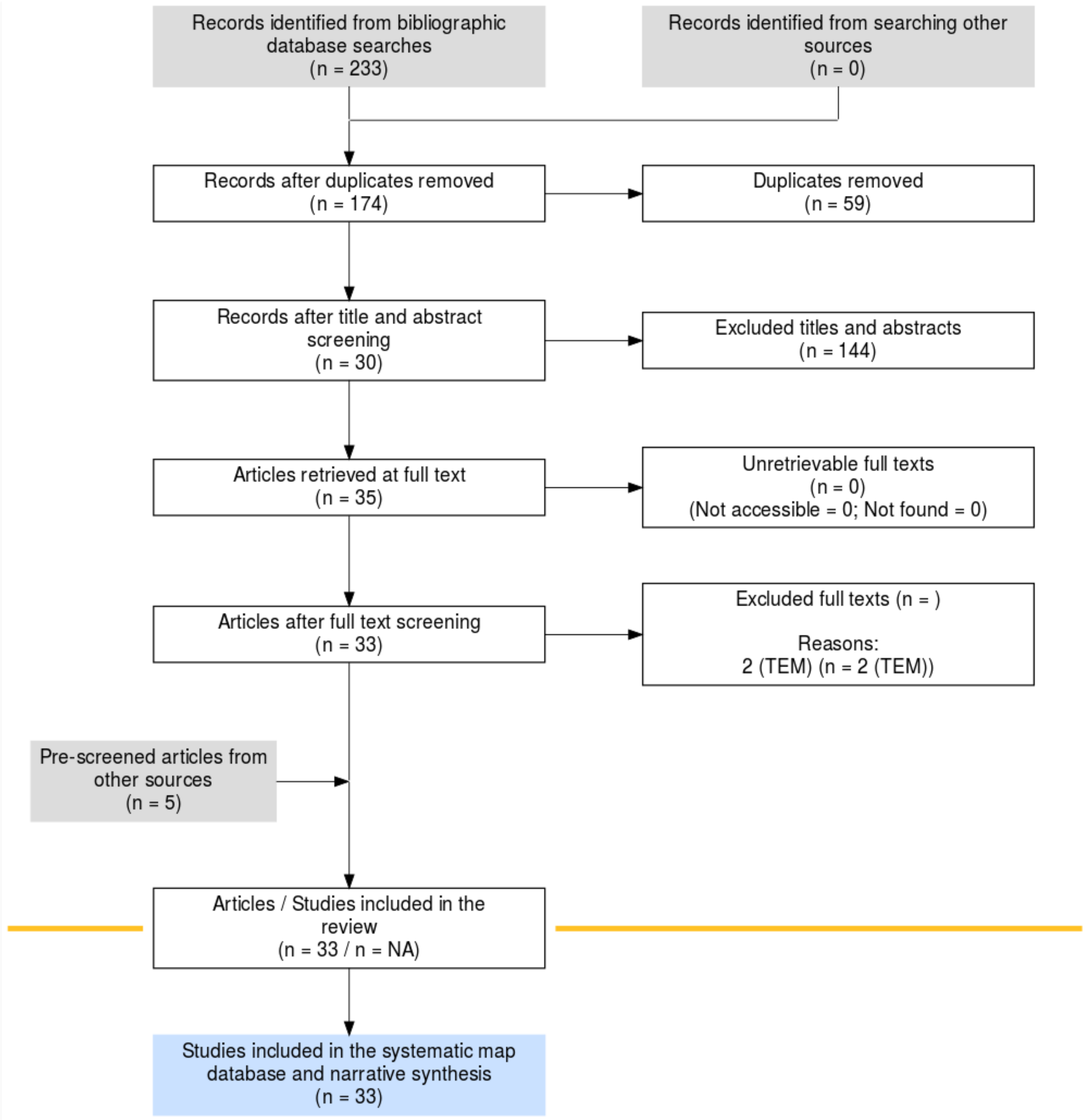

Figure S2. ROSES flow chart showing the results of application of the systematic protocol for reviewing literature.

Supplemental Information S1

Pilot procedure for development of systematic review methodology

1. Scoping phase

We conducted scoping searches in Scopus on title, abstract and keywords by combining terms with Boolean operators based on search phrases initially identified by the research team and

further refined through an iterative testing procedure. Based on this pilot, missing terms and relevant synonyms were added to the search strategy (e.g. disease “white plague”) forming the final list of search terms.

2. Screening phase

Criteria initially developed based on the research questions, and tested during a pilot on titles and abstracts of 25 randomly selected articles from the final merged reference list using Rayyan. Initial inclusion criteria were that the title and abstract must clearly state that the study is relevant to hard corals, a white disease and use of histological analysis. Studies were excluded if they likely referred to disease types not involving white disease (e.g. growth anomaly). For inclusion, the studies also must be available in English and have undergone peer-review. Any identified studies with insufficient information to be conclusively excluded based on the eligibility criteria were forwarded to the next phase. The pilot screening stage was validated by two independent reviewers (EA and CEP) and studies included were compared to determine whether the reviewers agreed with respect to inclusion criteria. Based on the pilot phase inclusion and exclusion criteria were amended with additional terms, for example the addition of exclusion of studies that were reviews of literature only and studies referring to other disease types such as black band and yellow band disease.

3. Data extraction

For each of the publications included in this study we systematically recorded information (i.e. data extraction) through filling in a pre-coded data sheet in Google Forms (see below) for general data extraction. The functioning of the datasheet was piloted on randomly selected 10 articles.

Final pre-filled data extraction form and form for critical appraisal of methodology can be found below.

Systematic review of histological methods for white diseases in hard corals: Data extraction

Charlotte Page and Ella Anderson

1. Title

2. Publication_year

3. DOI
   Starting with 10. Leave empty if no DOI available.

4. Study_first_author (Last name)

5. Exclusion_reason

Tick all that apply.

- [ ] NA - study included for data extraction
- [ ] No full-text available
- [ ] Full text in English
- [ ] Study does not consider hard coral
- [ ] Study does not consider a white disease
- [ ] Study does not conduct histological analysis
- [ ] Other/Unclear
- [ ] Study is a review
- [ ] Study refers to a tissue loss disease that does not have white signs

6. Comment for exclusion reason

____

7. Study_type

Tick all that apply.

- [ ] Experimental (lab)
- [ ] Field
- [ ] Experimental & Field
- [ ] Other: _______________________________

8. Comment for study type

____

9. List the genera that were examined for histology and associated disease (i.e. Acropora, white syndrome; followed by the number of species examined per genus. Separate Genus, disease with ;) e.g. Acropora, white syndrome; 6; Montipora, white syndrome; 3

10. Time of sample collection (if available dates)

11. Study location (Country OR Ocean if field) - if relevant, NA if no country

12. Sites sampled (separate by ;) - if relevant, NA if no site

13. Environmental parameters recorded

Tick all that apply.

- [ ] Temperature
- [ ] Water quality
- [ ] Depth
- [ ] Other (specify below)
- [ ] N/A

14. Comment for other parameters recorded

15. What sample types were involved in the study for histology analysis.

Tick all that apply.

- [ ] Apparently healthy (healthy)
- [ ] Apprently healthy tissue from a diseased colony
- [ ] Diseased (lesion border)
- [ ] Diseased (lesion)
- [ ] Other/comment

16. Comment on types of samples

17. What other methods were utilised?

Tick all that apply.

- [ ] Underwater surveys (Prevalence, Severity, Multi-species or Single-species)
- [ ] Photophysiology (Pulse Amplitude Modulated Fluorometery, Dark adapted yield, Quantum yield)
- [ ] Host physiology (comment specifics below)
- [ ] Other endosymbiont physiology (comment specifics below)
- [ ] Microbial (comment specifics below)
- [ ] Other: ____________________

18. Comment on other methods utilised

19. Number of biological replicates per sample group (i.e. the number of colonies sampled)

____

20. Number of technical replicates (if applicable) (i.e. the number of fragments from each colony)

____

21. Number of histology sections examined per technical replicate (per fragment) (if applicable)

____

22. Are the methods for histological analysis stated?

Mark only one oval.

- ⬭ Yes
- ⬭ No (other reference given - specify below)

23. Citation for methods

____
____
____
____
____

24. What was used to fix coral samples?

Mark only one oval.

- Paraformaldehyde
- Z-fix
- Other: ____________

25. What were the samples enrobed in?

Mark only one oval.

- Agarose
- NA
- Other: ____________

26. What was used in decalcification?

Mark only one oval.

- Formic acid/formaldehyde
- EDTA
- HCl
- Other: ____________

27. What were samples embedded in?

Mark only one oval.

- Paraffin
- Other: ____________

28. What was the thickness of the tissue sections (μm)?

____

29. What stain was used on the coral tissue?

Tick all that apply.

- [ ] Hematoxylin and eosin
- [ ] Giemsa
- [ ] Phloxine B
- [ ] Trichrome
- [ ] FISH
- [ ] Apoptag/ISEL
- [ ] Alcian blue
- [ ] Other: ____________________

30. Comment

____
____
____
____
____

31. What imaging devises were used?

Tick all that apply.

- [ ] Light microscope
- [ ] Transmission electron microscope
- [ ] Confocal microscope
- [ ] Other: ____________________

32. Focus of histological analysis

Tick all that apply.

- [ ] Disease-causing agent (also called aetiology)
- [ ] Tissue condition
- [ ] Reproduction
- [ ] Other

33. Comment on focus of histological analysis

34. Were the data collected qualitative and/or quantitative?
Purely descriptive = Qualitative; Generates data = Quantitative; Semi-quantitative = Scores (e.g. 1-5 with definitions)

Tick all that apply.

- [ ] Qualitative
- [ ] Quantitative
- [ ] Semi-quantitative (provide scoring system below)

35. If quantitative or semi-quantitative, what/how were things measured?

36. If semi-quantitative, how many levels of scoring were applied? (e.g. 5)

37. What was examined in the histology sections?

Tick all that apply.

- [ ] Tissue fragmentation
- [ ] Wound repair
- [ ] Necrosis
- [ ] Swelling and/or lysis of host cells
- [ ] Cellular integrity of host
- [ ] Cellular integrity of endosymbiont
- [ ] Endosymbiont abundance
- [ ] Gonad development (oocytes and spermaries)
- [ ] Count of oocytes (specify per polyp or unit area)
- [ ] Programmed Cell Death (also called Apoptosis)
- [ ] Associated organisms (bacteria)
- [ ] Associated organisms (ciliates)
- [ ] Associated organisms (fungi)
- [ ] Associated organisms (helminths)
- [ ] Associated organisms (sponge)
- [ ] Associated organisms (other, specify below)
- [ ] Other: ______________________

38. If the paper provides definitions (i.e. visual descriptions) of the above terms, specify here). Separate definitions by ;. E.g. Necrosis = xxxxx;

39. Comment (specifics associated with each of the above).

40. What was the main finding of this paper?

This content is neither created nor endorsed by Google.

Google Forms

Systematic review of histological methods for white diseases in hard corals: Appraisal of methods reporting

Charlotte Page and Ella Anderson

1. Title

2. Publication_year

3. DOI
Starting with 10. Leave empty if no DOI available.

4. Study_first_author (Last name), gender (he, she, they - as specified through a google search with a focus on pronouns he/him, she/her, they/them), institution country (at time of publishing)

5. Study_second_author (Last name), gender (he, she, they - as specified through a google search with a focus on pronouns he/him, she/her, they/them), institution country (at time of publishing)

6. Study_last_author (Last name), gender (he, she, they - as specified through a google search with a focus on pronouns he/him, she/her, they/them), institution country

____

7. Lead contact: Are details specified for a lead contact?

Tick all that apply.

- [ ] Yes
- [ ] No

8. Materials availability: Are the raw data released with this paper?

Mark only one oval.

- ( ) Yes
- ( ) No
- ( ) Contact author

9. Materials availability: Is the code released with the paper?

Mark only one oval.

- ( ) NA
- ( ) Yes
- ( ) No
- ( ) Contact author

10. Is another paper cited in replacement of providing adequate details of the methods used?

Mark only one oval.

- Yes
- No

11. Subject details: Are the species studied listed?

Mark only one oval.

- Yes
- No (genus level only)
- Other (mix of species and genus only)

12. Subject details: If the study is experimental, are full details on aquaria maintenance (including temperature, flow, open/closed/semi-open), acclimation times, feeding. provided? 0 - No details, 1 - Some details but not enough to repeat the experiment, 2 - All details have been specified

Tick all that apply.

- [ ] NA
- [ ] 0
- [ ] 1
- [ ] 2

13. Methods: What information is provided on when samples were sampled?

Mark only one oval.

- Date (d/m/y)
- Month
- Not specified
- Time frame
- Year

14. Methods: How is the sampling location specified?

Tick all that apply.

- [ ] Country
- [ ] GPS coordinates
- [ ] Site (reef-level)
- [ ] Ocean
- [ ] No location specified

15. Methods: What information on sample sizes was provided? Tick if sample sizes for each group were specified within the study.

Tick all that apply.

- [ ] Biological (individual colony)
- [ ] Technical (within a colony)
- [ ] Sections (per fragment)

16. Methods: Were any of the following procedures applied during tissue evaluation?

Tick all that apply.

- [ ] Masking (during scoring of samples)
- [ ] Randomisation (during processing of samples)

17. Methods: Are repeatable definitions of scoring criteria or tissue pathologies stated?

Mark only one oval.

- ( ) Yes
- ( ) No
- ( ) For some terms, but not all

18. Methods: Are the names of statistical tests applied stated? NA if no statistics are used.

Mark only one oval.

- NA
- Yes
- No

19. Methods: If statistics are used, is there explanation of how significance is determined?

Mark only one oval.

- NA
- Yes
- No

This content is neither created nor endorsed by Google.

Google Forms

References

1. Marcus E. A STAR is born. Cell. 2016;166(5):1059–60.

2. Gibson-Corley KN, Olivier AK, Meyerholz DK. Principles for valid histopathologic scoring in research. Veterinary pathology. 2013;50(6):1007–15.

3. Aeby G, Ushijima B, Bartels E, Walter C, Kuehl J, Jones S, et al. Changing stony coral tissue loss disease dynamics Through Time in *Montastraea cavernosa*. Front Mar Sci. 2021;8.

4. Ainsworth TD, Kramasky-Winter E, Loya Y, Hoegh-Guldberg O, Fine M. Coral disease diagnostics: what's between a plague and a band? Applied and Environmental Microbiology. 2007;73(3):981–92.

5. Eaton K, Landsberg J, Kiryu Y, Peters E, Muller E. Measuring stony coral tissue loss disease Induction and Lesion Progression Within Two Intermediately Susceptible Species, *Montastraea cavernosa* and *Orbicella faveolata*. Frontiers In Marine Science. 2021 Sep 8;08.

6. Gignoux-Wolfsohn SA, Precht WF, Peters EC, Gintert BE, Kaufman LS. Ecology, histopathology, and microbial ecology of a white-band disease outbreak in the threatened staghorn coral *Acropora cervicornis*. Dis Aquatic Org. 2020;137(3):217–37.

7. Landsberg JH, Kiryu Y, Peters EC, Wilson PW, Perry N, Waters Y, et al. Stony coral tissue loss disease in Florida Is Associated With Disruption of Host–Zooxanthellae Physiology. Front Mar Sci. 2020;7.

8. Miller MW, Lohr KE, Cameron CM, Williams DE, Peters EC. Disease dynamics and potential mitigation among restored and wild staghorn coral, *Acropora cervicornis*. PeerJ. 2014;2014(1).

9. Peters EC. A survey of cellular reactions to environmental stress and disease in Caribbean scleractinian corals. Helgolander Meeresunters. 1984;37(1–4):113–37.

10. Pollock FJ, Wada N, Torda G, Willis BL, Bourne DG. White syndrome-affected corals have a distinct microbiome at disease lesion fronts. Appl Environ Microbiol. 2017;83(2).

11. Rodriguez-Martinez R, Banaszak A, McField M, Beltran-Torres A, Alvarez-Filip L. Assessment of *Acropora palmata* in the Mesoamerican Reef System. PLOS ONE. 2014 Apr 24;9(4).

12. Sere M, Tortosa P, Chabanet P, Turquet J, Quod J, Schleyer M. Bacterial Communities Associated with *Porites* white patch syndrome (PWPS) on Three Western Indian Ocean (WIO) Coral Reefs. PLOS ONE. 2013 Dec 31;8(12).

13. Work TM, Russell R, Aeby GS. Tissue loss (white syndrome) in the coral *Montipora capitata* is a dynamic disease with multiple host responses and potential causes. Proceedings of the Royal Society B: Biological Sciences. 2012;279(1746):4334–41.

14. Work TM, Aeby GS, Hughen KA. Gross and Microscopic Lesions in Corals from Micronesia. Vet Pathol. 2016;53(1):153–62.

15. Work TM, Aeby GS. Pathology of tissue loss (white syndrome) in *Acropora sp*. corals from the Central Pacific. J Invertebr Pathol. 2011;107(2):127–31.

16. Work TM, Rameyer RA. Characterizing lesions in corals from American Samoa. Coral Reefs. 2005;24:384–90.

17. Howells EJ, Vaughan G, Work TM, Burt J, Abrego D. Annual outbreaks of coral disease coincide with extreme seasonal warming. Coral Reefs. 2020;39:771–81.

18. Andersen SB, Vestergaard ML, Ainsworth TD, Hoegh-Guldberg O, Kühl M. Acute Tissue Death (white syndrome) Affects the Microenvironment of Tabular *Acropora* Corals. Aqua Biol. 2010;10(1):99–104.

19. Smith HA, Conlan JA, Pollock FJ, Wada N, Shore A, Hung JYH, et al. Energy depletion and opportunistic microbial colonisation in white syndrome lesions from corals across the Indo-Pacific. Sci Rep. 2020;10(1).

20. Ainsworth TD, Kvennefors EC, Blackall LL, Fine M, Hoegh-Guldberg O. Disease and cell death in white syndrome of acroporid corals on the Great Barrier Reef. Mar Biol [Internet]. 2007;151(1):19–29.

21. Sweet MJ, Croquer A, Bythell JC. Experimental antibiotic treatment identifies potential pathogens of white band disease in the endangered Caribbean coral *Acropora cervicornis*. Proc R Soc B Biol Sci. 2014;281(1788).

22. Ainsworth TD, Kvennefors EC, Blackall LL, Fine M, Hoegh-Guldberg O. Disease and cell death in white syndrome of acroporid corals on the Great Barrier Reef. Mar Biol. 2007;151(1):19–29.
